# A coupled model between circadian, cell-cycle, and redox rhythms reveals their regulation of oxidative stress

**DOI:** 10.1038/s41598-024-66347-9

**Published:** 2024-07-05

**Authors:** Kosaku Masuda, Takeshi Sakurai, Arisa Hirano

**Affiliations:** 1 Institute of Medicine, University of Tsukuba, Tsukuba, Ibaraki 305-8575 Japan; 2 International Institute for Integrative Sleep Medicine (WPI-IIIS), University of Tsukuba, Tsukuba, Ibaraki 305-8575 Japan; 3 Life Science Center for Tsukuba Advanced Research Alliance, University of Tsukuba, Tsukuba, Ibaraki 305-8577 Japan

**Keywords:** Cell cycle, Circadian clock, Multicellularity, Oxidative stress, Redox rhythm, Synchronization, Dynamic networks, Oscillators, Computational models, Cell division, Circadian rhythms

## Abstract

Most organisms possess three biological oscillators, circadian clock, cell cycle, and redox rhythm, which are autonomous but interact each other. However, whether their interactions and autonomy are beneficial for organisms remains unclear. Here, we modeled a coupled oscillator system where each oscillator affected the phase of the other oscillators. We found that multiple types of coupling prevent a high H_2_O_2_ level in cells at M phase. Consequently, we hypothesized a high H_2_O_2_ sensitivity at the M phase and found that moderate coupling reduced cell damage due to oxidative stress by generating appropriate phase relationships between three rhythms, whereas strong coupling resulted in an elevated cell damage by increasing the average H_2_O_2_ level and disrupted the cell cycle. Furthermore, the multicellularity model revealed that phase variations among cells confer flexibility in synchronization with environments at the expense of adaptability to the optimal environment. Thus, both autonomy and synchrony among the oscillators are important for coordinating their phase relationships to minimize oxidative stress, and couplings balance them depending on environments.

## Introduction

Many organisms have three biological rhythms with an approximately 24-h cycle: the transcription-translation feedback loop (TTFL) of clock genes (hereafter referred to as the circadian clock), the cell cycle, and the redox rhythm. The circadian clock influences the periodic gene expression patterns of multiple clock genes, such as *Bmal1* and *Period1–3*, in mammals; thus, the circadian clock phase can be represented by the expression level of these genes^1^. Many physiological circadian rhythms of organisms, such as sleep–wake pattern, body temperature rhythm, and endocrine rhythms, are also driven by the circadian clock^2,3^. The cell cycle is a series of events that take place during cell division^4^ and is divided into four active phases, G1, S, G2, and M, responsible for cell growth, DNA synthesis, protein synthesis, and cell division, respectively, and a resting phase, G0. While some studies have reported that the circadian clock and cell cycle are interconnected^5–7^, cell division is still observed in organisms lacking clock genes^5^ and circadian rhythms occur in non-dividing cells, such as neurons or senescent cells, indicating that cell cycle is gated by, but exists in isolation from the circadian clock and vice versa^8,9^. The redox rhythm refers to the oscillations in the oxidation and reduction mechanisms in cells, represented by the level of peroxiredoxins (Prxs) in cells^10–12^. The redox rhythm is present in various organisms and has been observed even in mouse and human red blood cells, which lack nuclei (i.e., TTFL is absent) and do not undergo cell division^13–15^. Therefore, redox rhythms can be considered independent of the circadian clock and cell cycle. However, these rhythms interact with each other, as reactive oxygen species (ROS) are generated in response to behavioral activities in animals or photosynthesis activity in plants, and the redox rhythm is altered by mutations in the circadian clock genes^15–19^. Additionally, ROS generation affects both the cell cycle and circadian rhythm^15,16^. Therefore, although the redox rhythm is autonomous, it interacts with the other two oscillators.

The circadian resonance hypothesis states that the circadian clock provides a survival advantage to organisms by synchronizing their circadian rhythms with environmental rhythms^20–22^. For instance, in photosynthetic unicellular eukaryotes, cell division occurs at night, when the photosynthetic oxidative stress is minimized^23^. Of note, the loss of the circadian clock input to the cell cycle decreases cell proliferation^5,23^. In other words, there may be a time-dependent tolerance to oxidative stress in organisms during the cell cycle, and the circadian clock may mediate these phase relationships. However, to elucidate the phase relationship between the circadian clock, cell cycle, and redox rhythm, it is necessary to measure these three rhythms in a time series at a single-cell level. Additionally, it is experimentally difficult to artificially and independently control each rhythm and its interactions. Furthermore, higher organisms consist of many cells and each cell has autonomous rhythms and interactions with other cells^24–26^. The synchronization rate among cells affects the entrainment of cell population with environmental cycles^27–29^, which is why multicellular organisms have more complex oscillator dynamics.

In the present study, to determine whether the autonomy and synchrony of the circadian clock, cell cycle, and redox rhythm are beneficial for cells, we evaluated the relationship between these three oscillators by modeling their network. We combined a previously proposed coupled oscillator model for the circadian clock and cell cycle^30^ with a mathematical model for the redox rhythm^31^. We evaluated cellular fitness by synchronizing the oscillators in terms of cellular damage by oxidative stress. Our results indicate that moderate coupling brings the cell cycle and redox rhythm into a proper phase relationship and minimizes cell damage caused by oxidative stress. Furthermore, by extending the coupled oscillator model to a multicellular system, we also demonstrated the advantage of multicellularity in adapting to different environments.

## Results

### Modeling the coupling among three biological oscillators

**Figure 1:**
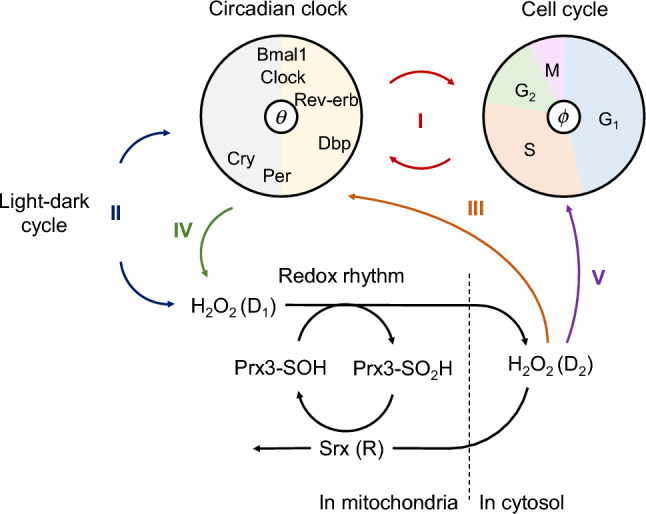
Schematic diagram of the three coupled oscillator model. Each numbered arrow represents a coupling between the respective oscillators.

We proposed a coupled oscillator system, in which each oscillator affected the phase of the other oscillators (Fig. 1). In this model, we used phase oscillator models for the circadian clock and cell cycle, in which the dynamics of these two oscillators was represented only by their phase, i.e., circadian time and cell-cycle phase, based on a previous study^30^. The phase of each rhythm is defined by a value from 0 to 2π during one cycle. The phase of the circadian clock at circadian time (CT) 0 (approximately the peak time of *Bmal1* expression) was defined as *θ* = 0, and the phase of the cell cycle at the end of M phase was defined as *ϕ* = 0. For the redox rhythm, we used a mathematical model developed in another previous study^31^. This model describes the dynamics of H_2_O_2_ produced in mitochondria as well as that of Prx and sulfiredoxin (Srx), which are involved in H_2_O_2_ degradation. The cellular H_2_O_2_ amounts show a rhythmic pattern in approximately 24-h periods. Although these models assume the cells to be mammalian, each element of the model is expected to be ubiquitous across many organisms.

In the proposed model (Fig. 1), we assumed the following interactions between each oscillator.

#### (I) Coupling of the circadian clock and cell cycle

Previous studies have modeled the relationship between circadian rhythms and the cell cycle, examining their dynamics in detail^30^. Previous models have assumed unilateral coupling between the two, but because there are various theories regarding this^6–9,32,33^, we assumed that they interact with each other. Although the coupling function between the circadian clock and cell cycle has been obtained in previous studies, for simplicity, the shape of the coupling function was assumed to be a sine function. Previous studies have shown that the peak of *Rev-erb-α* expression appears approximately 4 h after cell division^30^. Since the peak expression time of *Rev-erb-α* is approximately CT6, the circadian rhythm and cell cycle were assumed to be synchronized so that the phase difference between them is 0.1 × 2π (Fig. S1a,b).

#### (II) H_2_O_2_ generation and entrainment of circadian clock by light stimulation

While light stimulation generates H_2_O_2_ in the cell, it also entrains the circadian clock. Here, we assumed that light intensity varies sinusoidally in a time-dependent manner mimicking the environmental 12–12 h light–dark cycle and that H_2_O_2_ in mitochondria (*D*_1_) is generated in proportion to the light intensity. The circadian clock is assumed to change its phase according to the phase response curve (PRC), which is a summary of the phase responses to the stimulation at each phase. Light stimulation is assumed to entrain the circadian clock to CT6 (the center of daytime) via signals from the suprachiasmatic nucleus (SCN) in mammals^34^. For simplicity, the shape of the PRC was assumed to be a sine function (Fig. S1c). In coupling (II), the amplitude of PRC of the circadian clock for light stimulation and the H_2_O_2_ production rate by light were fixed, while the amplitude of the light intensity was variable. The average light intensity with and without light stimulation was set as 0.

#### (III) Phase shift of circadian clock by H_2_O_2_

Because H_2_O_2_ can induce a phase response in the circadian clock^35,36^, H_2_O_2_ rhythm produced in the cytosol (*D*_2_) should entrain the circadian clock. Based on the PRC measured in a previous study^35^, we assumed that its shape is a sine function (Fig. S1d) and defined that H_2_O_2_ entrains the phase of the circadian clock to CT14. If the effect of H_2_O_2_ is proportional to *D*_2_, the phase of the circadian clock will be constantly stimulated, regardless of rhythm variations in H_2_O_2_ concentration. Therefore, the strength of H_2_O_2_ effect on the circadian clock was considered proportional to the rhythmic component of H_2_O_2_, i.e., the H_2_O_2_ level minus the average H_2_O_2_ level (${D}_{2}-\overline{{D }_{2}}$).

#### (IV) H_2_O_2_ production associated with circadian activity

H_2_O_2_ level in the entire cell changes with circadian period, and the total H_2_O_2_ level (*D*_1_ + *D*_2_) reaches its peak at approximately CT12 in the mouse liver^37^. Our simulation also revealed that the time lag between H_2_O_2_ production and the peak H_2_O_2_ level (*D*_1_ + *D*_2_) is approximately 4 h in the redox rhythm model (Fig. S2). Therefore, we assumed circadian rhythm-associated H_2_O_2_ production to peak at CT8 (Fig. S1e). The amount of H_2_O_2_ production was assumed to vary sinusoidally throughout the day, and the daily amount of H_2_O_2_ production was assumed to be constant.

#### (V) Delay in cell cycle due to oxidative damage

High H_2_O_2_ concentrations cause DNA damage and cell cycle arrest, as repair of damaged DNA is necessary for cell cycle progression. Moderate H_2_O_2_ transiently increases the percentage of cells in the G2/M phase at approximately 24 h after H_2_O_2_ stimulation; however, after 48 h, many cells are arrested in the G1 phase^38^. This suggests that H_2_O_2_ causes a cell cycle delay before the G2/M phase. The DNA damage caused by H_2_O_2_ is thought to be proportional to the amount of H_2_O_2_ and DNA. In the cell cycle, the amount of DNA is doubled in the S phase and maintained until the end of the M phase. Therefore, we predicted that the delay in the cell cycle caused by H_2_O_2_ is larger during this period and set the value of *ϕ* as π at the beginning of the S phase when the increase in DNA content begins. For simplicity, we assumed that the cell cycle is delayed in proportion to *D*_2_ with a constant coefficient from the S phase to the end of the M phase (π ≤ *ϕ* < 2π) (Fig. S1f).

### Effects of coupling among the three oscillators

First, we checked the effect of the couplings (I–V defined in Fig. 1) on the dynamics of the three oscillators. At least two coupling factors were necessary for the three oscillators to synchronize. Because the coupling between the circadian clock and cell cycle (I) has already been analyzed in detail previously^30^, we assumed that it always exists, and the remaining coupling factors were added individually.

**Figure 2:**
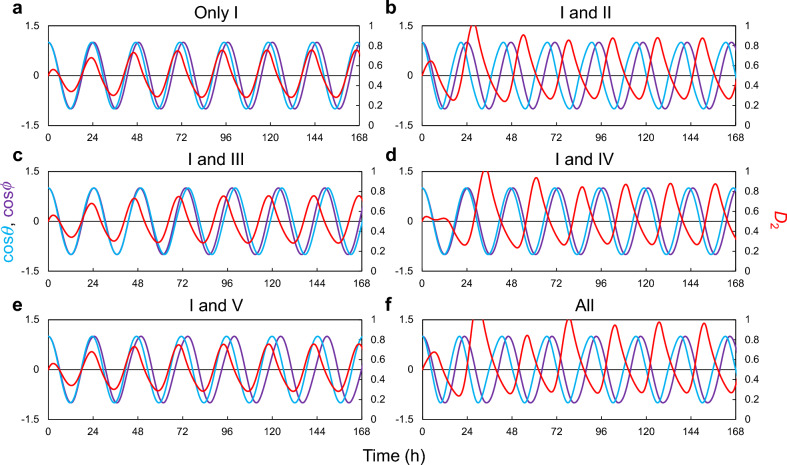
Rhythms of three oscillators with individual coupling factors. *θ* = *ϕ* = 0 (rad) and *D*_1_ = *D*_2_ = *R* = 0.5 at *t* = 0 (h). Coupling (I) was applied in all conditions (**a**–**f**), and the other couplings (II–V) were applied individually in (**b**–**e**) and simultaneously in (**f**). The parameters of the couplings for each condition are described in Table S1. cos*θ* and cos*ϕ* are shown on the left axis, and *D*_2_ on the right axis.

When only the circadian clock and cell cycle (I) were coupled, they were synchronized, and the peak of the cell cycle (M phase) was slightly behind the peak of the circadian rhythm (CT0) (Fig. 2a). However, since the redox rhythm was not coupled to the circadian clock or cell cycle, the phase difference remained unchanged from its initial value, indicating that the redox cycle operates independently. When one of the coupling factors (II–V) was added, the redox rhythm synchronized with the circadian rhythms and cell cycle (Fig. 2b–e). The phase relationships between the redox rhythm and the other rhythms were similar in all conditions. In particular, the cell cycle and redox rhythm were almost always in the opposite phases when at least two coupling factors were applied (Fig. 2b–e, Fig. S3). When all coupling factors were applied simultaneously, the phase relationships closely resembled those observed when each factor was applied individually (Fig. 2f). We evaluated the effect of the initial phase on the stabilized phase relationship after the transient period. When all couplings were applied simultaneously, the stabilized phase relationship was not substantially affected (Supplementary text 1; Fig. S4). However, when only two couplings (I + II, III, IV, or V) were applied in the model, the phase relationship was relatively unstable and fluctuated from the initial phases of the clock and cell cycle (Fig. S5). The phase differences remained relatively constant with respect to the strength of the coupling factor, when the coupling was sufficiently strong in each condition (Fig. S3).

### Circadian resonance in the three-oscillator model

**Figure 3:**
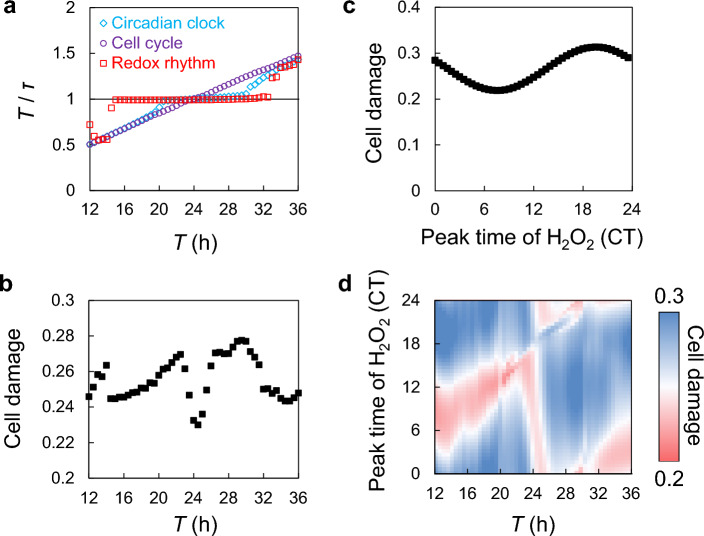
The effect of entrainment of circadian oscillators on cell damage. (**a**) Synchronization of the three biological oscillators under periodic environmental cycles. *T* and *τ* indicate the period of environmental cycle and each oscillator, respectively. When the *T*/*τ* is close to 1, the biological rhythm and environmental cycles are synchronized. (**b**) Cell damage against the period of environmental cycle. (**c**) Cell damage against the peak time of H_2_O_2_ production. (**d**) Change in cell damage against the period of environmental cycle and the peak time of H_2_O_2_ production.

Our model suggests that each coupling factor operates to prevent the overlap of the H_2_O_2_ peak with the M phase. Since radiosensitivity is highest in the M phase, we can assume that DNA damage due to oxidative stress is also highest in the M phase^39^. Moreover, we assumed that the sensitivity to H_2_O_2_ changes in a sinusoidal manner with a peak at the M phase. The maximum and minimum values of sensitivity were set as 1 and 0, respectively. Thus, the sensitivity to H_2_O_2_ is described as *S*(*ϕ*) = (1 + cos*ϕ*)/2. We calculated an index of cell damage, which is the average value of H_2_O_2_ amount *D*_2_ multiplied by the H_2_O_2_ sensitivity *S*(*ϕ*). In each condition, *D*_2_ and cos*ϕ* were obtained at each time as shown in Fig. 2, so cell damage was calculated by averaging the values of *D*_2_*S(*ϕ*) at all times. A higher index value indicates a greater H_2_O_2_ toxicity to the cells. Due to slight variations in the phase relationship among the three oscillators depending on the initial phases of the circadian clock and cell cycle (Supplementary text 1; Fig. S4), we present the mean value of cell damage for conditions starting from each initial phase of the circadian rhythm and cell cycle; however, the influence of the initial value appears to be limited. We evaluated cell damage under the environmental cycle (light cycle in this model) with a different period (Fig. 3a,b). We applied only the coupling factors (I) and (II) to consider the effect of synchronization with the environment. A ratio of the periods of environment (*T*) to each biological rhythm (τ) close enough to 1 indicates that each rhythm is entrained with the environment (Fig. 3a). All three biological rhythms could be entrained with environmental cycles with 24-h period, which is close to the free-running periods of the three internal rhythms. However, the range in which the redox rhythm and the circadian clock can be entrained with the environmental cycle was wider than that for cell cycles, because the redox rhythm and circadian clock are directly affected by the environmental cycles via coupling (II). Cell damage was minimized when the environmental period was approximately 24 h, in which all three oscillators could be entrained with the environment and synchronized with each other (Fig. 3b). Even when the three oscillators were synchronized, cell damage varied because the phase changed with the environmental period, and the most appropriate phase relationship was achieved at *T* = 24 h. This result indicates that the proposed model reproduced the circadian resonance phenomena, meaning that entrainment of the internal circadian rhythm with the environmental cycle is advantageous for the survival of organisms. We assumed a sinusoidal function for the PRC for light stimulus, but experimentally measured PRC includes a dead zone, where circadian rhythm exhibits no phase response. However, similar results were obtained even after assuming PRC with the dead zone for modeling (Fig. S6). Because previous studies have reported synchronization between circadian rhythm and cell cycle over a longer period^30^, we quantified the cell damage with stronger coupling of (I) and found similar results (Fig. S7). We then examined the effects of the circadian clock dysfunction (clock gene knockout; KO), i.e., lack of the coupling involved in the circadian clock (I–IV) (Fig. S8). Although some circadian rhythms, such as locomotor activity, are still weakly observed under a light–dark cycle in clock gene KO mice owing to the light masking effect^40^, we assumed that the couplings (I) to (IV) involve outputs from TTFL, which are largely lost after clock gene KO. The clock gene KO increased cell damage in both the presence and absence of coupling (V), suggesting that the circadian clock mediates the appropriate phase distribution of other rhythms to minimize cellular stress, including oxidative stress. This indicates that the potential cell damage is greater in clock gene-deficit organisms, which is consistent with the reports that the aging process appears faster in clock gene-KO animals^41^.

We also evaluated the cell damage when the peak of H_2_O_2_ production generated by the circadian rhythm was shifted (Fig. 3c). For this purpose, we considered only couplings (I) and (IV) and varied the phase of coupling (IV). In the condition without any perturbation, the peak time of H_2_O_2_ production was CT8 (Fig. 2d) and cell damage was lowest, indicating that maintaining a proper phase relationship between the circadian rhythms and H_2_O_2_ production can reduce cell damage (Fig. 3c). This result may explain the mechanism underlying the acceleration of the aging process when the circadian and activity rhythms are misaligned because of shift work or time-restricted feeding^42–44^. Furthermore, we evaluated the change in cell damage in response to changes in the peak time of H_2_O_2_ production and the period of the environmental cycle (Fig. 3d). When the period of the environmental cycle is 23–25 h, the timing of H_2_O_2_ production peak advanced as the period lengthened. In contrast, in other conditions (< 23-h or > 25-h environmental cycle), the timing of H_2_O_2_ production peak was delayed as the period lengthened. This suggests that when the environmental cycle deviates from a 24-h cycle owing to conditions such as shift work, oxidative stresses may be reduced by altering the timing of certain activities, such as eating or exercise. Even when the circadian rhythms are not synchronized with the environment with a period < 20 h or > 30 h (Fig. 3a), cell damage can be minimized if the peak time of H_2_O_2_ production is optimized (Fig. 3d).

The survival rate of cultured cells treated with H_2_O_2_ has been reported to depend on the circadian time, indicating that cellular H_2_O_2_ sensitivity or anti-oxidative rhythms may be clock dependent^45^. However, we observed similar results for circadian time in this study (Supplementary text 2; Fig. S9) to that when cell damage was defined by the phase of cell and redox cycles (Fig. 3). Therefore, our findings indicate that maintaining an optimal alignment between biological rhythms and environmental cycles is crucial for reducing cellular damage caused by oxidative stress.

### Adverse effects of strong coupling

**Figure 4:**
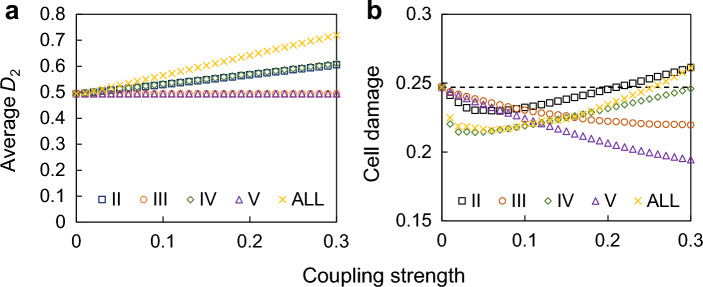
The cell damage depends on the coupling strength. (**a**) Average H_2_O_2_ level in cytosol (*D*_2_). (**b**) Cell damage against the coupling strength. Coupling (I) was applied in all conditions, and the other couplings were applied individually (II–V) or simultaneously (ALL). The strength of coupling (I) was the same as described in Fig. 2, and the strength of other couplings was varied. The ratio of the strength of each coupling in ALL was equal to that shown in Fig. 2f.

Next, to evaluate the effect of coupling strength on H_2_O_2_ levels in cytosol (*D*_2_) and cell damage (Fig. 4), we combined coupling (I) with the other couplings, as shown in Fig. 2b–e. We fixed the strength of (I) while changing the strengths of the other couplings (II–V). For the couplings (III) and (V), cell damage decreased with increasing coupling strength. For couplings (II) and (IV), it decreased at low coupling strengths. However, because the average concentration of *D*_2_ increased with increasing coupling strength of (II) and (IV), the cell damage increased at the high coupling strength. These results indicate that the optimal coupling strength that minimize oxidative stress is neither weak coupling (completely independent oscillators) nor very strong coupling. Similar results were obtained when the phase of the circadian time was used as an indicator of cellular damage (Fig. S9d).

**Figure 5:**
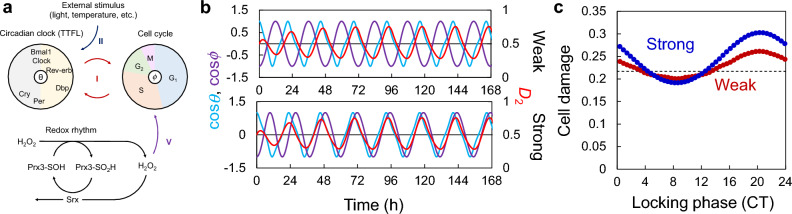
Indirect effects of environmental perturbations through strong coupling on cellular damage. (**a**) A model when circadian rhythms are disturbed. (**b**) The dynamics of three oscillators with weak (top) and strong (bottom) coupling (I) under environmental disturbance to the circadian rhythms. cos*θ* and cos*ϕ* are shown on the left axis, and *D*_2_ on the right axis. (**c**) Changes in cell damage for phase differences between redox and circadian rhythm with weak and strong coupling. Locking phase is the phase difference between the redox and circadian rhythms when the circadian clock synchronizes with the environment, i.e., the circadian time when the H_2_O_2_ concentration peaks.

Besides H_2_O_2_ and light, the circadian clock is synchronized with various other environmental factors, such as temperature and endocrine signals^46–48^. Consequently, if there is a stronger synchronizing factor than H_2_O_2_, the circadian clock phase can be transiently disturbed by it. To test this, we only applied a periodic environmental stimulus to the circadian clock as a clock perturbation signal (Fig. 5a). For simplicity, we assumed that the circadian clock receives environmental signals and the cell cycle receives signals from the circadian clock and redox rhythms. We then changed the strength of coupling (I) and timing of clock disturbance and evaluated changes in cellular damage depending on the phase difference (locking phase) between the circadian clock and redox rhythm when the circadian clock was synchronized with the environment. Here, we define the coupling as “weak” when the circadian rhythm and cell cycle can desynchronize due to disturbance, and “strong” when they maintain synchronization even in the presence of the disturbance. Even when environmental cycles disturb the circadian rhythms, the cell cycle and H_2_O_2_ rhythm can maintain an appropriate relationship with a weak coupling (I) (Fig. 5b). However, when coupling (I) is strong, the cell-cycle phase is affected by the disturbed circadian rhythm and the cell cycle cannot maintain an appropriate relationship with the redox rhythm. When the three rhythms were properly synchronized, the phase difference between the circadian clock and redox rhythm was around CT8 and there was no large difference in cell damage between strong and weak coupling conditions (Fig. 5c). However, when the stimuli caused a large phase shift in the circadian rhythm, where the H_2_O_2_ peaked around CT20, cell damage increased considerably only when the coupling was strong. These findings suggest that weak coupling between the circadian clock and the other oscillators contributed to not only synchronization with appropriate environments but also resistance to the disturbances.

**Figure 6:**
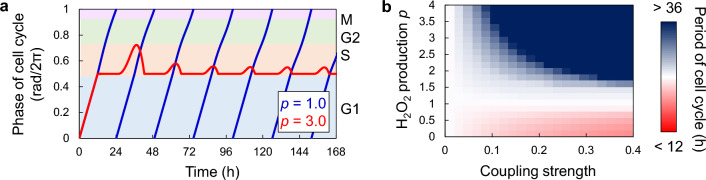
Cell cycle arrest under high H_2_O_2_ concentration. (**a**) Cell cycle progression under low (*p* = 1.0) and high (*p* = 3.0) H_2_O_2_ level. High H_2_O_2_ level caused cell cycle arrest. (**b**) Period of the cell cycle with respect to sensitivity of the cell cycle to H_2_O_2_ (coupling (V)) and changes in H_2_O_2_ production *p*.

When cells are severely damaged by oxidative stresses, the cell cycle is arrested^38^. This phenomenon was also observed in our proposed model (Fig. 6a), indicating that as the H_2_O_2_ concentration increased, cell cycle delay caused by H_2_O_2_ exceeded the rate of cell cycle progression, and the phase could not advance any further. Here, we considered only coupling (V) and varied the amount of H_2_O_2_ production. As the H_2_O_2_ concentration increased, the cell cycle period lengthened and eventually stopped completely (Fig. 6b). H_2_O_2_ concentration during cell cycle arrest depends on the sensitivity of the cell cycle to H_2_O_2_, which is represented as the coupling strength. Therefore, the cell cycle was not arrested even at high H_2_O_2_ concentrations when the cells were not sensitive to H_2_O_2_, whereas the cell cycle stopped even at small changes in H_2_O_2_ level when the cells exhibited high H_2_O_2_ sensitivity (Fig. 6b). These results suggest that the appropriate coupling strength between the redox rhythm and cell cycle is also important for maintaining a normal cell cycle and its arrest.

### Multicellular effect on the three oscillators

**Figure 7:**
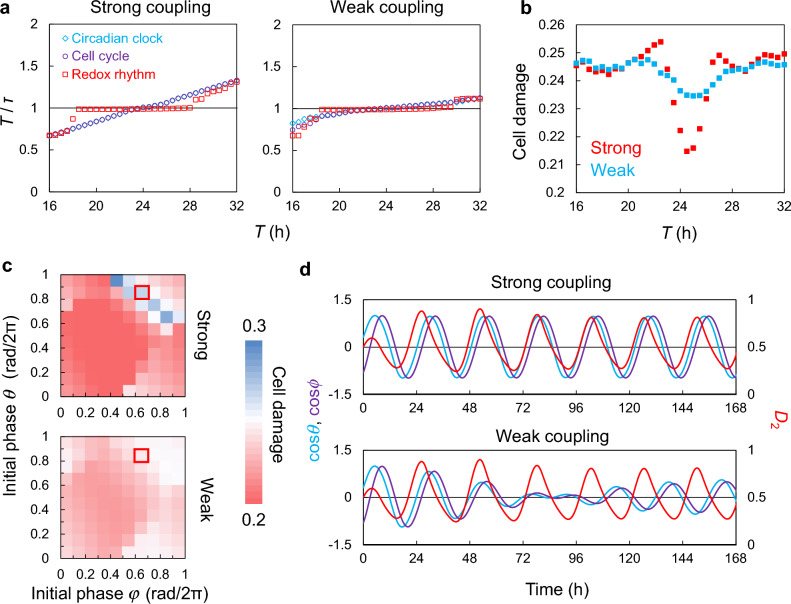
Relationship between intercellular coupling and entrainment of the three oscillators with environmental cycles. (**a**) Synchronization of the three oscillators with periodic environmental cycles in strongly and weakly coupled population. (**b**) Cell damage against the period of environmental cycles. (**c**) Cell damage for each initial phase at period *T* = 24 h. (**d**) Circadian rhythms in strongly and weakly coupled population under the unsuitable initial phases (surrounded by red box in (**c**)). cos*θ* and cos*ϕ* are shown on the left axis, and *D*_2_ on the right axis.

After demonstrating the significance of the autonomy of the three oscillators and their interactions at a single-cell level, we attempted to determine the influence of multicellularity on their synchronization. The frequency of the oscillators and the parameter that determines the frequency of the redox rhythm were assumed to follow a normal distribution with a standard deviation of 10%. The period of the circadian rhythm has been reported to vary from ± 1 to ± 3 h in cultured cells^49,50^. In the peripheral circadian clock, transforming growth factor beta mediates cell-to-cell coupling, and such growth factors are also involved in cell cycle regulation^25,51,52^. We assumed that the intercellular couplings of the circadian clock and cell cycle bring their phases closer to the average phase among cells. Moreover, because H_2_O_2_ permeates the cell membrane, we assumed that the H_2_O_2_ concentration in the cytoplasm approaches the average H_2_O_2_ concentration among cells. We first evaluated the entrainment to periodic environmental stimuli in the cell populations, where cells are strongly or weakly coupled with each other (Fig. 7a). Here, couplings (I) and (II) and intercellular couplings were considered, and intercellular coupling strength is defined as “weak” when the cells can desynchronize due to disturbance, and “strong” when they maintain synchronization despite disturbance. When the intercellular couplings were strong, the entrainable range of circadian rhythms and cell cycles was narrower than that of the redox rhythm (Fig. 3a). In contrast, when the intercellular couplings were weak, the three oscillators were synchronized when the period of stimuli cycle (*T*) was between 20 and 28 h, indicating that the entrainable period range was extended by weak synchronization among cells. The strong coupling contributed to lower cell damage than the weak intercellular coupling in a condition of *T* = 24 (h), where the three oscillators were well synchronized (Fig. 7b). However, in a condition of *T* = 22 or 27 (h), where the cell cycle in the strongly coupled population lost entrainment with the environment cycle, the cell damage was higher in the strongly coupled population than that in the weakly coupled population. This indicates that weak synchronization among cells moderates the change in the cell damage affected by changes in the period, instead of increasing the cell damage in the appropriate period (i.e., *T* = 24).

Further, we examined the cell damage when the initial phase of either the circadian rhythm or cell cycle was changed under an environmental cycle with a 24-h period (Fig. 7c). This can indicate the degree of change in cell damage when the phase relationship between the three oscillators is transiently disturbed. In the strongly coupled population, cell damage decreased when the three oscillators were in the appropriate phase relationship, whereas it increased when they deviated from the optimized phase relationship (Fig. 7c). However, in the weakly coupled population, cell damage was barely affected by the fluctuation in the phase of the cell cycle and circadian rhythms, suggesting that weakly coupled cells are more resistant to such perturbations compared with strongly synchronized cells. We compared the rhythms in the strongly and weakly coupled population at the initial phase when the cell damage was high in the strongly coupled population (Fig. 7d). In the strongly coupled population, the phase differences gradually changed, taking approximately 2 weeks to settle into the proper phase relationship. In contrast, in the weakly coupled population, the amplitude of the circadian rhythm and cell cycle was greatly reduced, and their phase quickly changed, taking less than 1 week to reach the optimized phase relationship to minimize cell damage. However, even when each oscillator reached an entrained state, the amplitude of the circadian rhythm and cell cycle in the weakly coupled population was smaller than that in the strongly coupled population because of the phase differences among cells. Similar results were obtained when the oxidative sensitivity was defined by the circadian clock phase (Fig. S10). We also examined the effect of multicellularity with weak and strong intercellular interaction on populations with couplings (III) to (V) (Figs. S11–S13). No significant changes in cell population with coupling (IV) between the two conditions (weak and strong cellular couplings) were observed. However, in populations with coupling (III) or (V), the variation in cell damage with respect to the initial phases of the circadian clock and cell cycle was greater in the strongly coupled population. Under conditions of large differences in oxidative stress between weak and strong intercellular couplings, the rhythms took longer to synchronize in the strongly coupled population, whereas they quickly converged to the proper phase relationship in the weakly coupled population by decreasing their amplitude (Figs. S11b and S13b). These results indicate that cellular synchrony and autonomy are a tradeoff between specialization and versatility in adapting to environmental cycles and that intercellular couplings balance them.

## Discussion

This study modeled the couplings between the circadian clock, cell cycle, and redox rhythm and evaluated their dynamics and physiological functions. The couplings among these oscillators regulate their phase relationship to avoid the overlap of the H_2_O_2_ peak with the M phase. Additionally, synchronization among oscillators and with the environmental cycle is important in maintaining these relationships. However, too strong coupling can increase cell damage and disrupt the rhythmicity of the cell cycle, when a transient perturbation occurs. Furthermore, the multicellularity simulation showed that synchrony and autonomy of cell population are a tradeoff between adaptability to a specific environment (i.e., *T* = 24 without phase perturbation) and adaptability to unstable environments (i.e., broader period of environmental cycle and/or phase perturbation). These results indicate that both synchrony and autonomy between the circadian clock, cell cycle, and redox rhythm have advantages and disadvantages and that an optimal balance between them for cell survival exists depending on the environment.

We made some assumptions in this study about the coupling between the three oscillators. However, the nature of these couplings is still hypothetical, and their actual existence requires experimental verification. Several studies have confirmed the existence of a coupling between circadian rhythms and the cell cycle^6–9,32,33^. Although we assumed mutual coupling, some studies have suggested a unilateral coupling from the cell cycle to the circadian rhythm^30^. However, even when a unilateral coupling is considered, the present results can be reproduced if either coupling (IV) or (V) exists (Fig. S14). Similarly, we obtained equivalent results when we assumed a simplified coupling function (Fig. 2) and a sine waveform coupling function (Fig. S15) between the cell cycle and H_2_O_2_ (V). Therefore, as long as the phase relationship between the oscillators is maintained, the shape of the coupling function may not have a large effect on the results. In Fig. 2, since we did not assume a direct input from the environmental cycle to the cell cycle, the cell cycle can be entrained by the environmental cycles only when the period of the cycle is close to 24 h. However, when the coupling between the circadian clock and the cell cycle is stronger, the range of synchronization becomes wider. Even in this case, the results are similar to those obtained when the coupling is weaker, indicating that entrainable range does not qualitatively affect results (Fig. S7). However, the synchronization of the cell cycle with the light–dark cycle in plants was reported^23^, while there are few results that have rigorously evaluated the entrainment of the cell cycle with environmental cycles without circadian-clock regulation. This is also true for the redox rhythm. More detailed study of how each independent rhythm synchronizes with the environmental cycles is needed in the future study. This study analysis primarily focused on individual couplings. Although many cells may exhibit multiple or all couplings simultaneously, the relationship between each coupling is expected to vary from cell to cell. Therefore, the conditions tested in this study can only be applied to specific cells or organisms and our model was constructed by aggregating the results of several studies, which may introduce some bias in the data underlying each assumption. In this study, the circadian rhythm and cell cycle models are based on NIH3T3 and U2OS cells, while the model of redox rhythm assumes mitochondria-rich organs, such as adrenal gland, heart, and brown adipose tissue. The coupling assumptions also include the results of liver and light entrainment via the SCN. It will be important to evaluate the effects of each rhythm and coupling under unified conditions to validate the model. In addition, a recent paper indicated that ROS levels peak in G2 and mitosis in cancer cells^53^. This result contradicts our results. However, the couplings assumed in the present model are based on non-cancer cells, and therefore pathological cells such as cancer cells, may exhibit different couplings or lose some couplings. There is another possibility that mitosis-derived ROS production peaking at M phase and the clock/redox rhythm-derived ROS production troughing at M phase occur independently. In this case, it may be reasonable to think that the couplings presented in this study work to avoid the overlap of mitosis-derived ROS production with the circadian clock- and redox rhythm-derived H_2_O_2_ peaks. In any case, future studies are needed to examine the nature of the rhythms and couplings in more types of cells. Although we used phase oscillators for the circadian clock and cell cycle here, detailed mathematical models of molecular mechanisms and their interaction have been described previously^33,54^. The mechanism by which DNA damage causes phase shifts in circadian rhythms has also been modeled^55,56^. The integration of these models may predict the dynamics of the three oscillators at the molecular level. Conversely, a simpler model can be constructed in which the redox rhythm can be described as a phase oscillator model like the circadian clock and cell cycle (Supplementary text 3; Fig. S16). The entrainment of oscillator is greatly affected when the amplitude of the oscillator can be significantly changed or not^57,58^. However, as shown in Fig. S16, similar results were obtained even when the redox rhythm was assumed as a phase oscillator, in which its amplitude does not change. Therefore, the effect of the amplitude changes at the single oscillator was not significant for the present results. A recent in silico study showed that the coupling between the circadian clock and the redox rhythm improves the stability of the rhythm, similar to our results^59^. However, that study also showed that the synchronization with the circadian clock is disturbed when the coupling between redox rhythms is too strong, suggesting the existence of an appropriate coupling strength from a different view than ours. In addition to the intracellular couplings, intercellular interactions have been studied for circadian rhythms^27,28,60,61^, and some efforts have been made to quantify the strength of intercellular couplings using experimental data and mathematical models^30,62,63^. Therefore, quantifying intracellular and intercellular couplings through experiments and mathematical analysis will allow more accurate prediction of the dynamics of the three oscillators.

We evaluated the effects of synchronization with the light cycles and the circadian rhythms on cells, and our results reinforced the circadian resonance phenomena. Therefore, our model could also be used to assess environmental suitability, such as the appropriate timing of eating or exercise for shift workers. Moreover, considering that H_2_O_2_ concentration is chronically increased in senescent cells^64^, our model also demonstrated that the reduced amplitude and phase shift of the circadian clock and cell cycle were observed, similar to senescent cells and aged mice^9,65^ (Fig. S17). Therefore, our model may also be useful for designing daily habits according to the circadian rhythms in older adults. Experimental investigation of the effects of multiple factors such as sleep^66,67^, diet^42–44,68^, exercise^18,69^, medications^70–72^, and aging^41–44,73^ on circadian rhythm and health is complex. Therefore, modeling the relationship between circadian rhythms and health/aging is crucial for designing lifestyle taking the circadian clock into account.

Overall, our results indicate the significance of the autonomy of biological rhythms and their synchronization with environmental and internal rhythms. Although most of the results are reproductions of circadian resonance phenomena, it is intriguing to note that cell damage may be altered by the relationship between activity peaks and environmental periods (Fig. 3d), and the strength of cell population coupling, which can also be interpreted as a model of central and peripheral clocks (Fig. 7). Since these three rhythms are ubiquitous in many organisms, the concepts presented in this study may be applicable to many organisms. Although we focused our analysis on oxidative stress, these results may be extrapolated to various biological rhythms (e.g., endocrine signals, glucose level, and oxygen concentration). However, the environment surrounding these rhythms may be actually more complex, and the present model only illustrates a part of them. It is essential to develop a system to simultaneously measure various biological rhythms in cells to validate the present results. Future studies analyzing the interactions with more elements may improve the precision of the model and deepen our understanding of the significance of biological rhythms in organisms.

## Methods

### Three coupled oscillator model

The simulation model is described as follows:

1 $$\frac{d\theta }{dt}=\omega +{f}_{\text{cel}\to \text{cir}}\left(\phi -\theta \right)+L{f}_{\text{light}\to \text{cir}}\left(\theta \right)+\left({D}_{2}-\overline{{D }_{2}}\right){f}_{\text{red}\to \text{cir}}\left(\theta \right),$$

2 $$\frac{d\phi }{dt}=\omega +{f}_{\text{cir}\to \text{cel}}\left(\theta -\phi \right)+\left({D}_{2}-\overline{{D }_{2}}\right){f}_{\text{red}\to \text{cel}}\left(\phi \right),$$

3 $$\frac{d{D}_{1}}{dt}=p+{k}_{\text{light}\to \text{red}}L-aA{D}_{1}-d{D}_{1}+{f}_{\text{cir}\to \text{red}}\left(\theta \right),$$

4 $$\frac{d{D}_{2}}{dt}=d{D}_{1}-e{D}_{2},$$

5 $$\frac{dR}{dt}=e{D}_{2}-qR,$$

6 $$A=\frac{bR}{bR+a{D}_{1}},$$

7 $$L=\text{cos}(\Omega t).$$

Here, $\theta$ is the phase of the circadian rhythm and $\phi$ is the phase of the cell cycle. $\theta =0$ was defined as CT0 (approximately the peak time of *Bmal1* expression), and $\phi =0$ was defined as the end of M phase. *ω* is the frequency of the circadian rhythm and cell cycle, and *ω* = 2π/24 (rad/h) in this model. $\Omega$ is the frequency of the light cycles. *D*_1_ and *D*_2_ represent the H_2_O_2_ concentration in the mitochondria and cytosol, respectively; *R* represents Srx in mitochondria; and *A* and *I* = 1 − *A* represent active/inactive Prx 3 (Prx3-SOH/Prx3-SO_2_H) in mitochondria, respectively. $\overline{{D }_{2}}$ is the average value of ${D}_{2}$ without coupling, which was 0.5 in this study. *a*, *b*, *c*, *d*, *e*, and *q* are parameters related to the redox rhythm, and their values were same as those used in a previous study (*a* = 1000, *b* = 2, *c* = 10,000, d = 0.2, e = 0.1, and *q* = 0.1). *p* is the amount of H_2_O_2_ produced, and *p* = 1.0 unless otherwise stated. *L* is a function of light intensity. The initial phase of the circadian rhythm and the cell cycle were set to each of 10 points every 0.2π from 0 (rad), i.e., $\left\{\theta \left(0\right),\phi \left(0\right)\right\}=\{2\pi *k/10, 2\pi *l/10\}$(*k* = 1, …,10 and *l* = 1, …, 10), and if not stated, the results are the average of all initial values. The initial values for the redox rhythm were set to ${D}_{1}={D}_{2}=R=0.5$.

The function representing each coupling is expressed as follows:

8 $${f}_{\text{cir}\to \text{red}}\left(\theta \right)={k}_{\text{cir}\to \text{red}}\text{cos} \left(\theta -\frac{8}{24}*2\pi \right),$$

9 $${f}_{\text{light}\to \text{cir}}\left(\theta \right)=-{k}_{\text{light}\to \text{cir}}\text{sin}\left(\theta -\frac{6}{24}*2\pi \right),$$

10 $${f}_{\text{red}\to \text{cir}}\left(\theta \right)=-{k}_{\text{red}\to \text{cir}}\text{sin} \left(\theta -\frac{14}{24}*2\pi \right),$$

11 $${f}_{\text{cel}\to \text{cir}}\left(\phi -\theta \right)={k}_{\text{cel}\to \text{cir}}\text{sin}\left(\phi -\theta -0.1*2\pi \right),$$

12 $${f}_{\text{cir}\to \text{cel}}\left(\theta -\phi \right)={k}_{\text{cir}\to \text{cel}}\text{sin}\left(\theta -\phi +0.1*2\pi \right),$$

13 $${f}_{\text{red}\to \text{cel}}\left(\phi \right)=\left\{\begin{array}{c}0\ \left( 0\le \phi <\pi \right),\\ -{k}_{\text{red}\to \text{cel}}\ \left(\pi \le \phi <2\pi \right),\end{array}\right.$$

where ${f}_{\text{A}\to \text{B}}$ and ${k}_{\text{A}\to \text{B}}$ represent the coupling functions and strengths from the rhythm A to rhythm B, and “cir,” “cel,” and “red” indicate circadian clock, cell cycle, and redox rhythm, respectively. The values of coupling strength used in each simulation are shown in Table S1. In Fig. 5, ${k}_{\text{light}\to \text{red}}=0$ and the peak time of *L* was changed.

The sensitivity to H_2_O_2_ is assumed to depend on the phase of cell cycle and is expressed as follows:

14 $$S\left(\phi \right)=\frac{\left(1+\text{cos}\phi \right)}{2}.$$

The average damage to the cells is represented by

15 $$\text{Cell damage}=\frac{1}{{t}_{\text{sim}}}{\int }_{0}^{{t}_{\text{sim}}}{D}_{2}(t)S(\phi \left(t\right))dt,$$

where ${t}_{\text{sim}}$ is a simulation time and ${t}_{\text{sim}}$ = 336 h in this study.

### Multicellular model

The multicellular model is described by the following equations:

16 $$\frac{d{\theta }_{j}}{dt}={\omega }_{\text{cir},j}+{f}_{\text{cel}\to \text{cir}}\left({\theta }_{j}\right)+L{f}_{\text{light}\to \text{cir}}\left({\theta }_{j}\right)+\left({D}_{2}-\overline{{D }_{2}}\right){f}_{\text{red}\to \text{cir}}\left({\theta }_{j}\right)+{f}_{\text{cir}\to \text{cir}}\left({\theta }_{j}\right),$$

17 $$\frac{d{\phi }_{j}}{dt}={\omega }_{\text{cel},j}+{f}_{\text{cir}\to \text{cel}}\left({\phi }_{j}\right)+\left({D}_{2}-\overline{{D }_{2}}\right){f}_{\text{red}\to \text{cel}}\left({\phi }_{j}\right)+{f}_{\text{cel}\to \text{cel}}\left({\phi }_{j}\right),$$

18 $$\frac{d{D}_{1,j}}{dt}=p+{k}_{\text{light}\to \text{red}}L-a{A}_{j}{D}_{1,j}-d{D}_{1,j}+{f}_{\text{cir}\to \text{red}}\left({\theta }_{j}\right),$$

19 $$\frac{d{D}_{2,j}}{dt}={d}_{j}{D}_{1,j}-e{D}_{2,j}+{f}_{\text{red}\to \text{red}}\left({D}_{2,j}\right),$$

20 $$\frac{d{R}_{j}}{dt}=e{D}_{2,j}-q{R}_{j},$$

21 $${A}_{j}=\frac{b{R}_{j}}{b{R}_{j}+a{D}_{1,j }},$$

22 $$L=\text{cos}(\Omega t).$$

Here, the subscript *j* indicates the value of the *j*th cell. The intercellular couplings are described by the following functions:

23 $${f}_{\text{cir}\to \text{cir}}\left({\theta }_{j}\right)=\frac{{k}_{\text{cir}\to \text{cir}}}{N}\sum_{k=1}^{N}\text{sin}({\theta }_{k}-{\theta }_{j}),$$

24 $${f}_{\text{cel}\to \text{cel}}\left({\phi }_{j}\right)=\frac{{k}_{\text{cel}\to \text{cel}}}{N}\sum_{k=1}^{N}\text{sin}({\phi }_{k}-{\phi }_{j}),$$

25 $${f}_{\text{red}\to \text{red}}\left({D}_{2,j}\right)=\frac{{k}_{\text{red}\to \text{red}}}{N}\sum_{k=1}^{N}\left({D}_{2,k}-{D}_{2,j}\right).$$

Here, *N* = 100 representing the cell number. The values of coupling strength used in each simulation are shown in Table S1. Initial phases in circadian clock and cell cycle are equal in all cells, respectively, and the initial phases were set to each of 4 points every π/2 from 0 (rad). The frequencies followed a normal distribution with mean 2π/24 and standard deviation of 2π/24 × 0.1. The initial values for the redox rhythm were set to ${D}_{1}={D}_{2}=R=0.5$ in all cells. In redox rhythm, only the parameter *d* was varied and assumed to follow a normal distribution with a mean of 0.2 and standard deviation of 0.02. The parameters were same as those used in the single-cell model. Cell damage at the population level was determined as the average of cell damage in all cells.

### Calculation of periods

The period of a circadian clock was defined by the following equation:

26 $${\tau }_{\theta }=\frac{2\pi {t}_{\text{sim}}}{\theta \left({t}_{\text{sim}}\right)-\theta \left(0\right)}.$$

The period of a cell cycle was calculated by the same equation.

For calculation of the period of a redox rhythm, the peak of *R* was defined as the point that satisfies the following equation:

27 $${R}_{l}>{R}_{l-1},{R}_{l}>{R}_{l+1},$$

where *l* indicates the *l*th data point. The period of the redox rhythm was calculated as

28 $${\tau }_{\text{red}}=\frac{{t}_{\text{last}}-{t}_{\text{first}}}{{N}_{\text{peak}}-1}.$$

Where ${t}_{\text{first}}$ and ${t}_{\text{last}}$ are the first and last peak, respectively, and ${N}_{\text{peak}}$ is the peak number. In the case of the cell population, the periods were obtained for the average rhythms of all cells.

### Supplementary Information

Supplementary Information.

## Data Availability

All data supporting the findings of this study are available within the paper and its Supplementary Information.
