# Supplementary material for: A coupled model between circadian, cell-cycle, and redox rhythms reveals their regulation of oxidative stress
Source: Sci Rep. 2024 Jul 5;14:15479. doi: 10.1038/s41598-024-66347-9 (PMC11226698; doi:10.1038/s41598-024-66347-9)
Supplement: Supplementary file 1 — Supplementary Information. [file 41598_2024_66347_MOESM1_ESM.docx]

**A coupled model between circadian, cell-cycle, and redox rhythms reveals their regulation of oxidative stress.**

Supplementary text 1–3

Figure S1–S17

Table S1

**Supplementary text 1. Effect of the initial phases on the dynamics of the three oscillators.**

We examined the effect of initial phases of the circadian clock and cell cycle on the synchronization of the three oscillators. When we assumed only coupling I, the final phase difference between the cell cycle and the redox rhythm was proportional to the initial value of the cell cycle (Figure S4). On the other hand, when one among coupling II to V was additionally applied, the phase difference was around π for most of the initial conditions, but some initial conditions resulted in deviated phase relationship, where phase difference between cell cycle and redox rhythms was close to 0 (2π). This may be because the initial phase difference was precisely at the point where the value of the coupling function was almost 0, resulting in minimal phase change from its initial value (Figure S5). However, when all couplings (I–V) were applied, the phase difference was converged to the same values in any initial condition. For assessing cellular damage, we calculated the mean value at the stabilized conditions starting from each initial phase (Figure 3).

**Supplementary text 2. Evaluation of cell damage based on circadian time.**

Although we assumed that sensitivity to oxidative stress increases during the M phase of the cell cycle, it is also possible to base sensitivity to H_2_O_2_ on circadian time, since the H_2_O_2_ peak in Figure 2 was in the opposite phase of the peak for the circadian clock. In fact, a cell line derived from mouse retina showed maximal survival rate in response to oxidative stress at CT16 (*45*). Therefore, we evaluated the cell damage under the same conditions, assuming a sine waveform with a maximum sensitivity to oxidative stress at CT4 (Figure S9a–c). Under the same conditions as in Figure 3ab, the cell damage was reduced when circadian and redox rhythms were synchronized but over a wider period range because the circadian rhythm and redox rhythm were synchronized over a wider range than the cell cycle. When the time of H_2_O_2_ production changed depending on the circadian clock, cell damage was minimized when H_2_O_2_ peaked around CT10, as shown in Figure 3c. However, when the environmental cycle and peak time of H_2_O_2_ production were varied simultaneously, as in Figure 4d, oxidative stress was lower over a wider period range than when oxidative stress was determined based on the cell cycle. This might be because light stimuli more strongly synchronize the circadian and the redox rhythms than the cell cycle, resulting in lower cell damage even when the peak time of H_2_O_2_ production changes. However, the range of peak time where cell damage was lower depended on the environmental cycle. These results confirm that even when the circadian time is used as an indicator of cell damage, the appropriate relationship between the biological rhythms and environmental cycles is important for reducing cell damage caused by oxidative stress.

**Supplementary text 3. Simplified model of redox rhythm.**

In the present study, the circadian clock and cell cycle were represented by a phase oscillator model consisting of a single variable, whereas the redox rhythm was represented by a model with multiple variables based on a previous study (*31*). However, the redox rhythm can also be expressed only in terms of phase (Figure S16a). In the simplified model, the phase *ψ* of the redox rhythm was expressed by the following equations:

| $\frac{d\psi}{dt}=\omega+L*f_{\mathrm{light}\to\mathrm{red}}\left( \theta\right)+f_{\mathrm{cir}\to\mathrm{red}}\left( \theta\right),$ | (S1) |
| --- | --- |
| $D_{2}=0.5+0.5\cos(\psi-\pi/3),$ | (S2) |
| $f_{\mathrm{light}\to\mathrm{red}}\left( \theta\right)=\sin(\psi-2\pi*0.35)-0.5\sin\left\{ 2\left( \psi-2\pi*0.35 \right) \right\}.$ | (S3) |

Here, $\psi=0$ was defined as the peak time of *D*_1_. We assumed that the peak time of *D*_2_ is delayed by 4 h from the peak time of *D*_1_. For the circadian clock and cell cycle, we used Eqs. (1), (2), and (8)–(13). Calculations of cell damage were based on Eqs. (14) and (15). Calculations of the period of *ψ* were based on Eq. (26). The initial phase of the circadian rhythm, the cell cycle, and the redox rhythm were set to each of 10 points every 0.2π from 0 (rad), and if not stated, the results are the average values in all conditions starting from each initial value. Since we assumed that the input to the redox rhythm is only H_2_O_2_, the phase oscillator model for the redox rhythm can be expressed using the PRC and H_2_O_2_ stimulation. We obtained the PRC in a simulation, in which we provided a pulsatile H_2_O_2_ input to the redox rhythms described as the multiple-variable model (Figure S16b). PRCs were obtained for the phase shift for stimuli at each phase in which the value of *p* was increased or decreased by 0.1 for 1 h using Eqs. (3)–(6). Simulations of three coupled phase oscillator model with all couplings confirmed that the peaks of cell cycle and H_2_O_2_ rhythms appear in an opposite phase (Figure S16c), similar to the results shown in Figure 2, wherein we used the multiple-variable model for the redox rhythms. The entrainment with light cycles and the circadian resonance phenomenon were also observed in the same condition as in Figure 3ab (Figure S16de). We also evaluated the relationship between the peak of H_2_O_2_ production and the cell damage and confirmed that cell damage was lowest at approximately CT8 (Figure S16f), which was also similar to the results in Figure 3c. Therefore, by simplifying the redox rhythm as a phase oscillator, the dynamics of the three oscillators can be treated as the dynamics of three coupled phase oscillators.


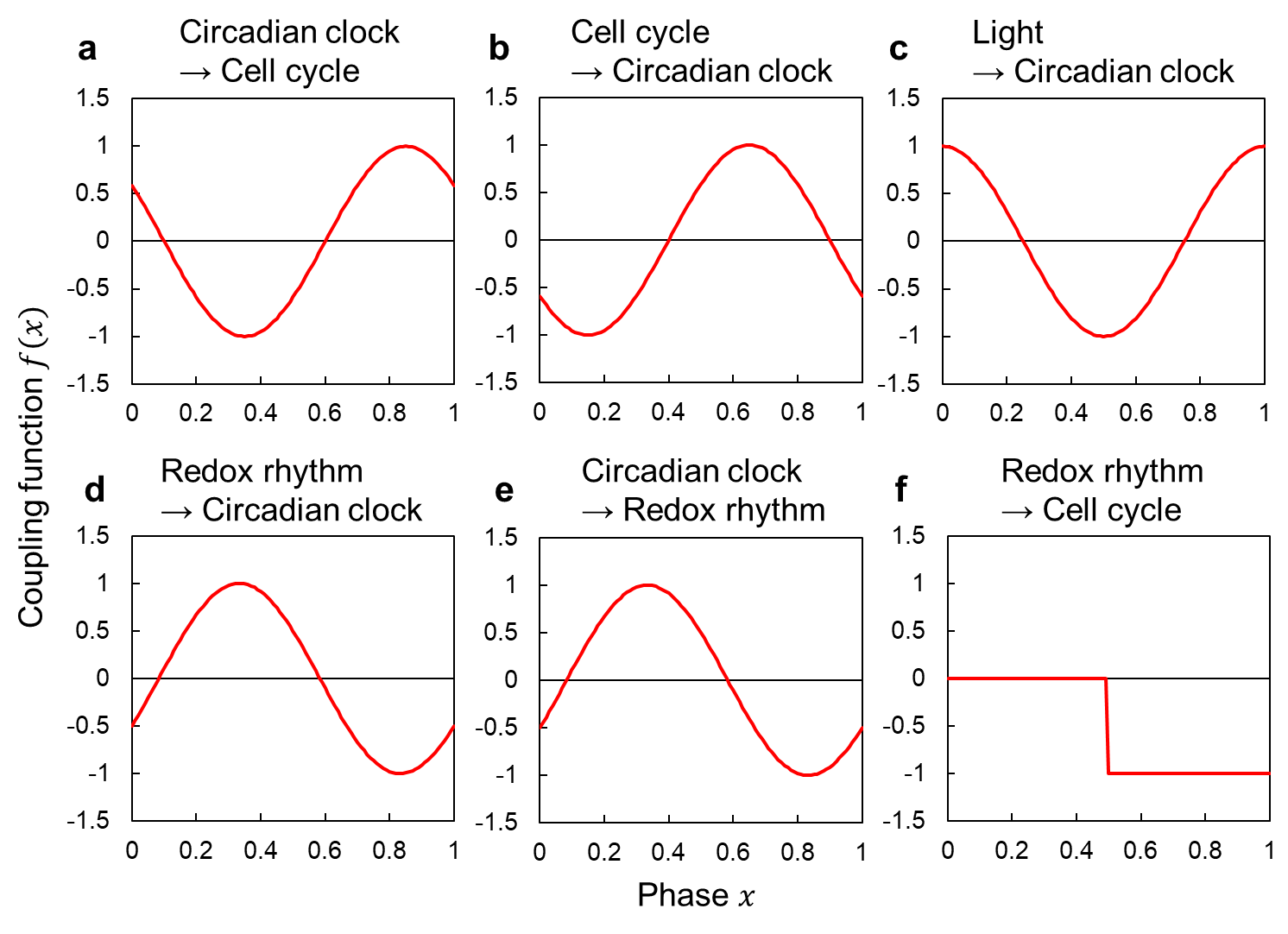


Figure S1. The shapes of the coupling functions. **a** $f_{\mathrm{cel}\to\mathrm{cir}}\left( \phi-\theta\right)$, **b** $f_{\mathrm{cir}\to\mathrm{cel}}\left( \theta-\phi\right)$, **c** $f_{\mathrm{light}\to\mathrm{cir}}\left( \theta\right)$, **d** $f_{\mathrm{red}\to\mathrm{cir}}\left( \theta\right)$, **e** $f_{\mathrm{cir}\to\mathrm{red}}\left( \theta\right)$, and **f** $f_{\mathrm{red}\to\mathrm{cel}}\left( \phi\right)$. (**a**) and (**b**) correspond to coupling (I), (**c**) to (II), (**d**) to (III), (**e**) to (IV), and (**f**) to (V). Coupling strength *k* = 1 in all panels.


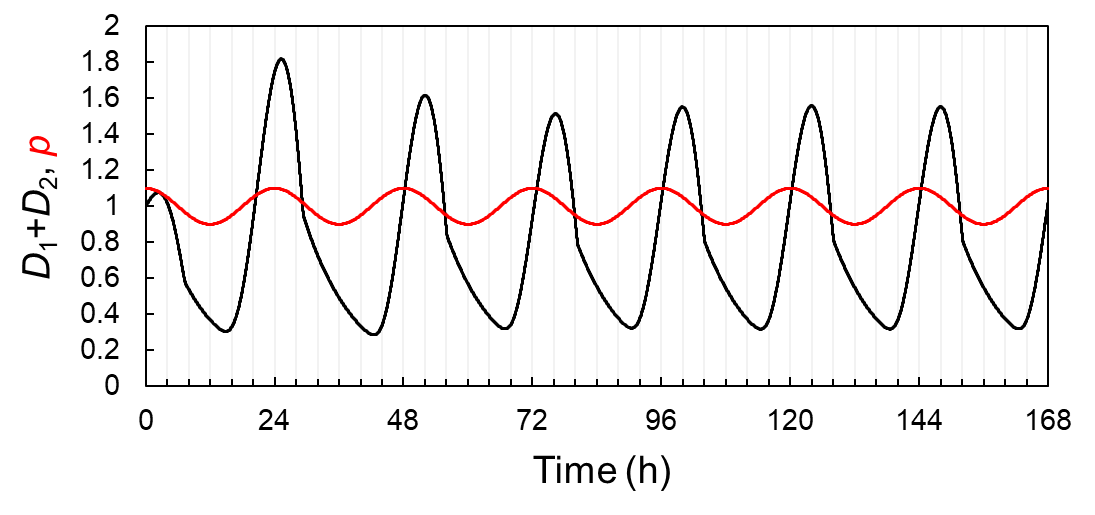


Figure S2. Time lag between the peaks of H_2_O_2_ production and H_2_O_2_ level in the cell. *D*_1_ + *D*_2_ (black line) indicates the total H_2_O_2_ level in the cell. *p* (red line) indicates the amount of H_2_O_2_ production. The simulation was performed in the model for redox rhythm without couplings with the circadian clock and cell cycle.


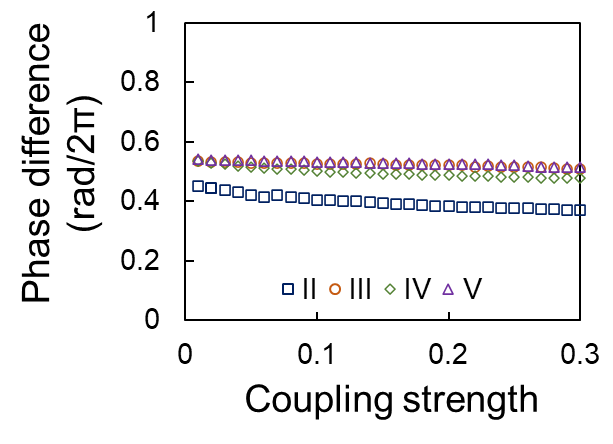


Figure S3. The phase difference between the cell cycle and redox rhythm.


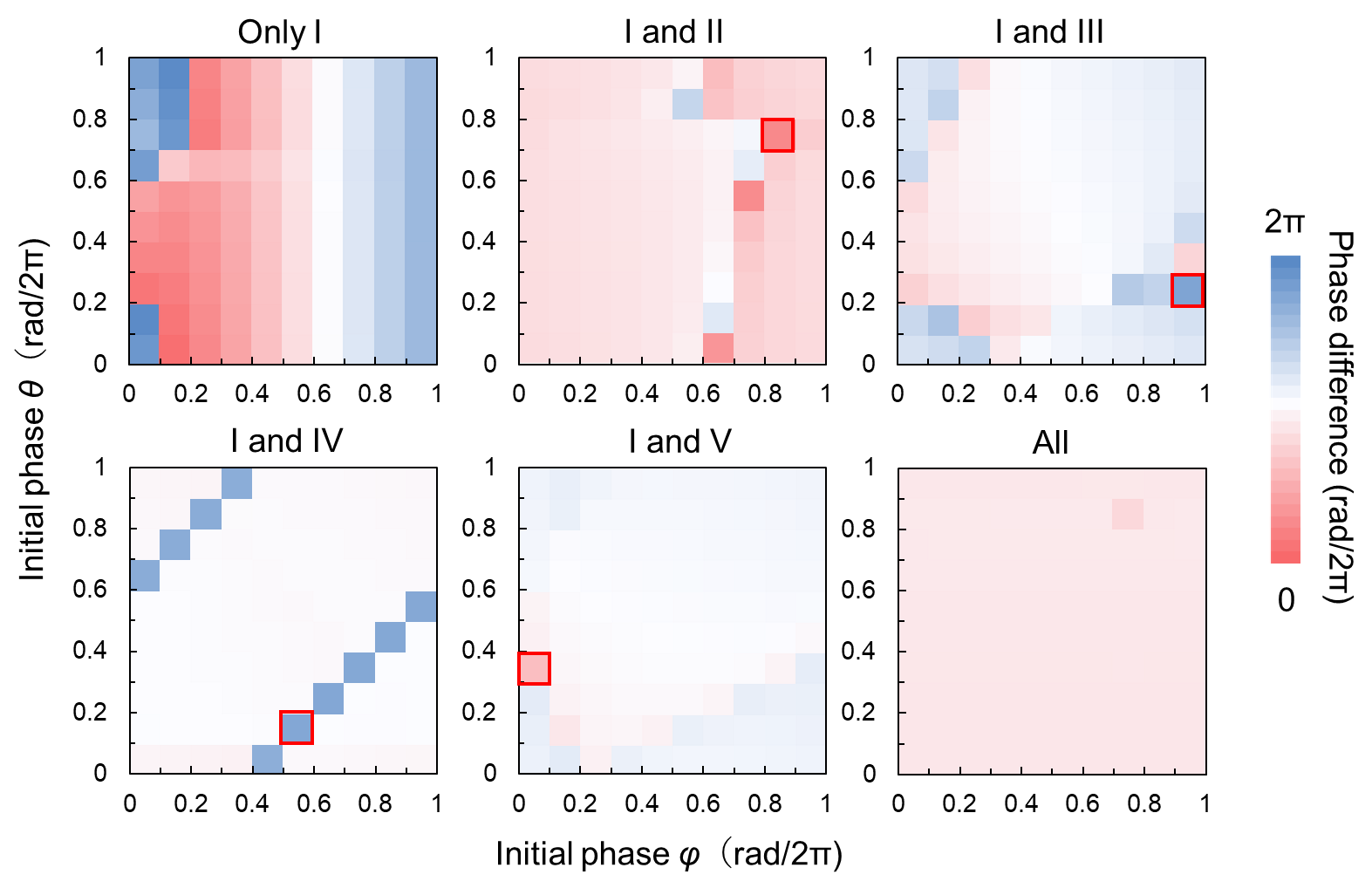


Figure S4. Effect of the initial phases of the circadian clock and cell cycle on the stabilized phase differences between the cell cycle and redox rhythm. The sets of initial phases resulting in the final phase difference mostly deviated from π and are marked with red boxes.


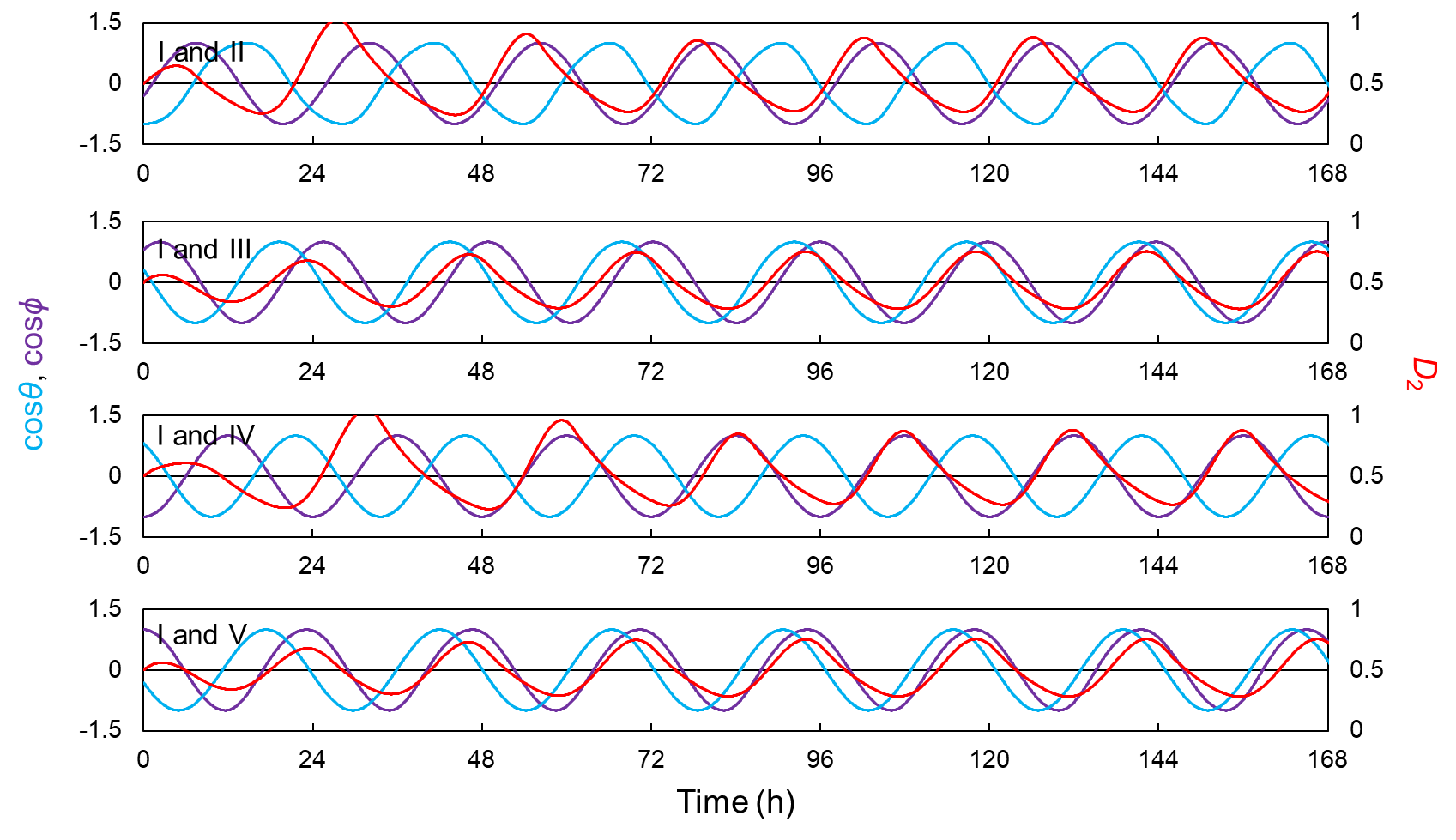


Figure S5. Rhythms of three oscillators at the initial phases where the rhythms are hardly synchronized. The sets of initial phases of the circadian clock and cell cycle are indicated by the red boxes in Figure S4.


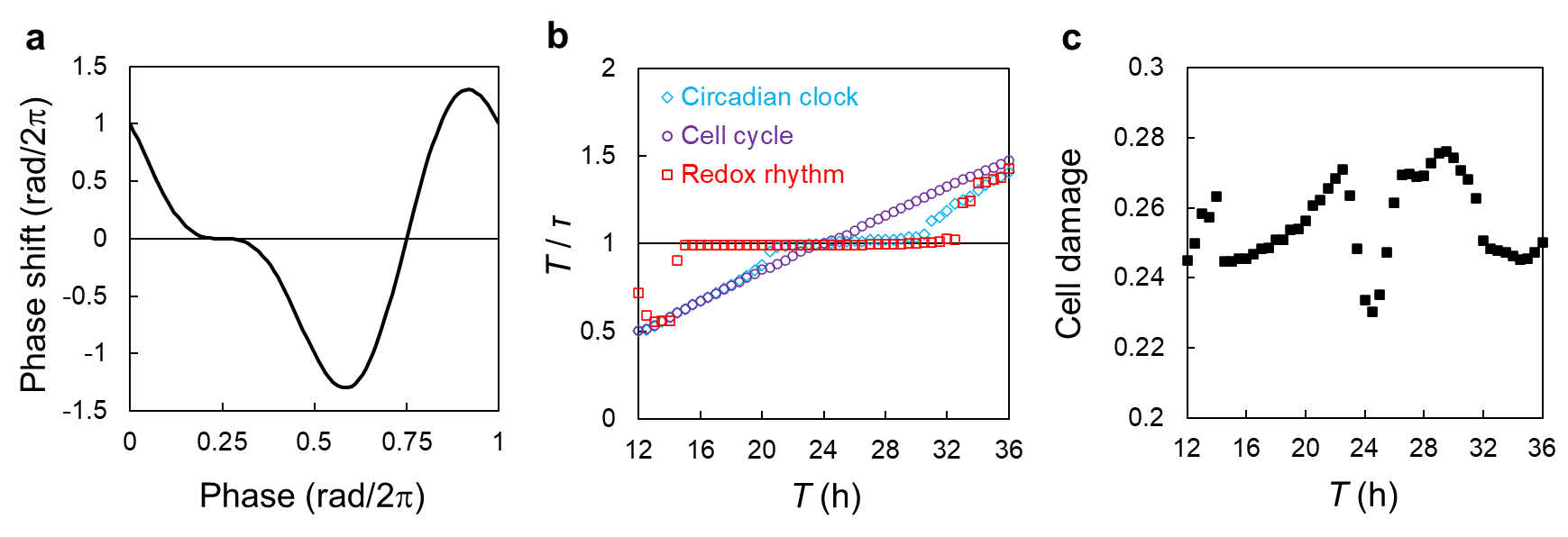


Figure S6. The effect of entrainment of circadian oscillators on cell damage when the PRC for light stimulus has a dead zone. **a** Phase response curve with a dead zone, the time windows where the clock does not show phase response to light. **b** Synchronization of the three biological oscillators under periodic environmental cycles. **c** Cell damage against the period of environmental cycle.


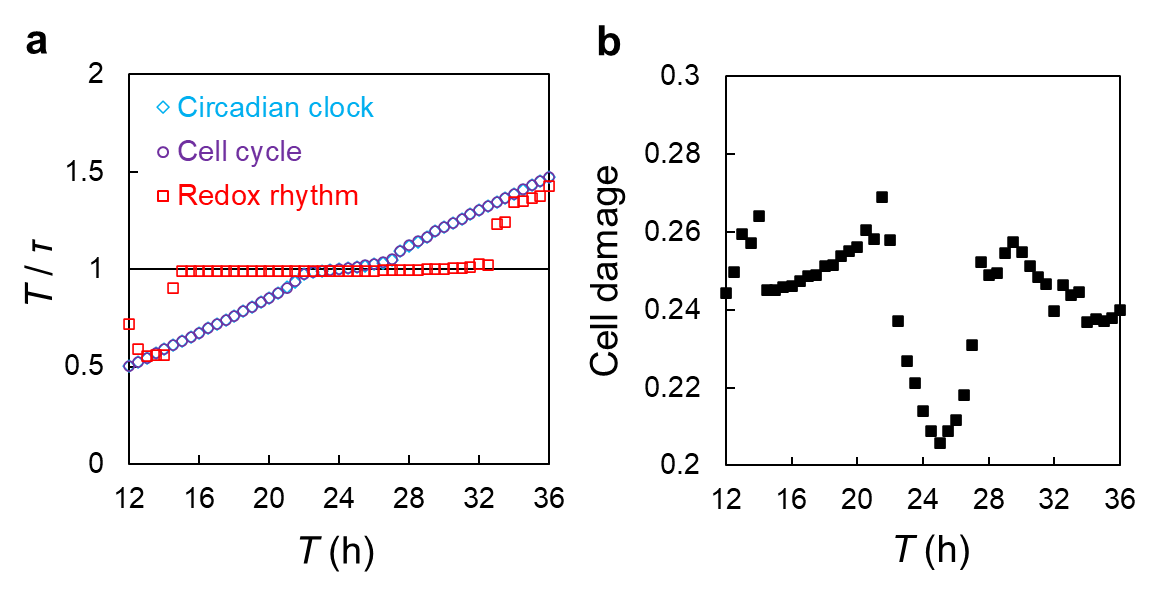


Figure S7. The effect of entrainment of circadian oscillators on cell damage when the coupling (I) is strong. **a** Synchronization of the three biological oscillators under periodic environmental cycles. **b** Cell damage against the period of environmental cycle.


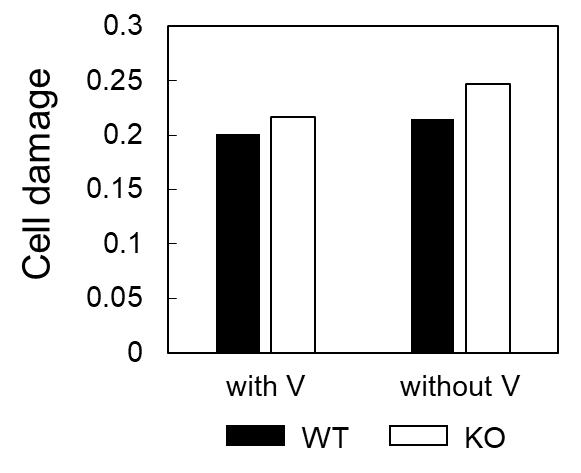


Figure S8. Cell damage in wild-type (WT) and clock gene knockout (KO) cells with and without coupling (V). Couplings (I–IV) are given in WT and not in KO.


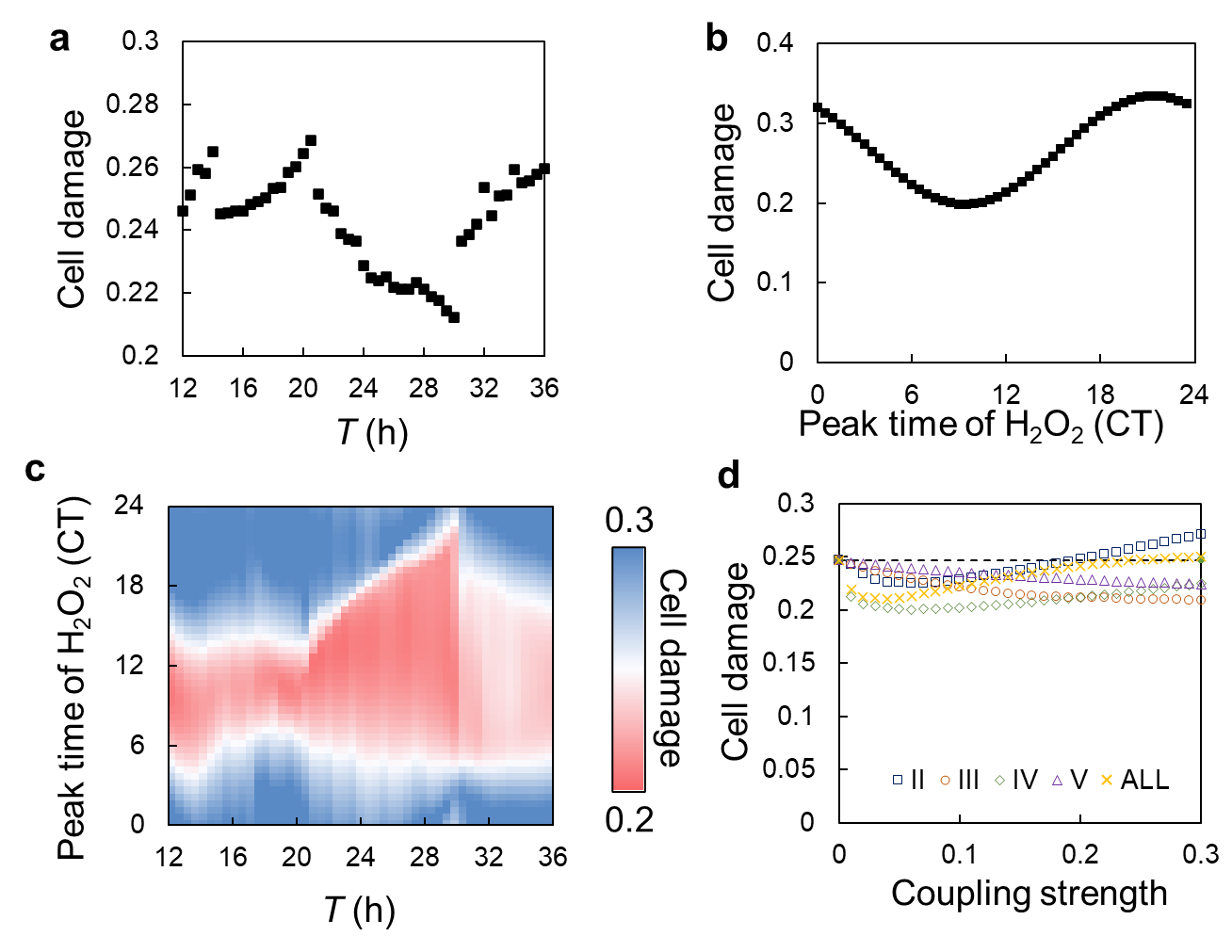


Figure S9. Cell damages calculated based on the phase of circadian clock. **a** Cell damage against the period of environmental cycle. **b** Cell damage against the peak time of H_2_O_2_ production. **c** Change in cell damage against the period of environmental cycle and the peak time of H_2_O_2_ production. **d** Cell damages depending on the coupling strength. The condition of couplings is the same as in Figure 3 in (a–c) and Figure 4 in (d).


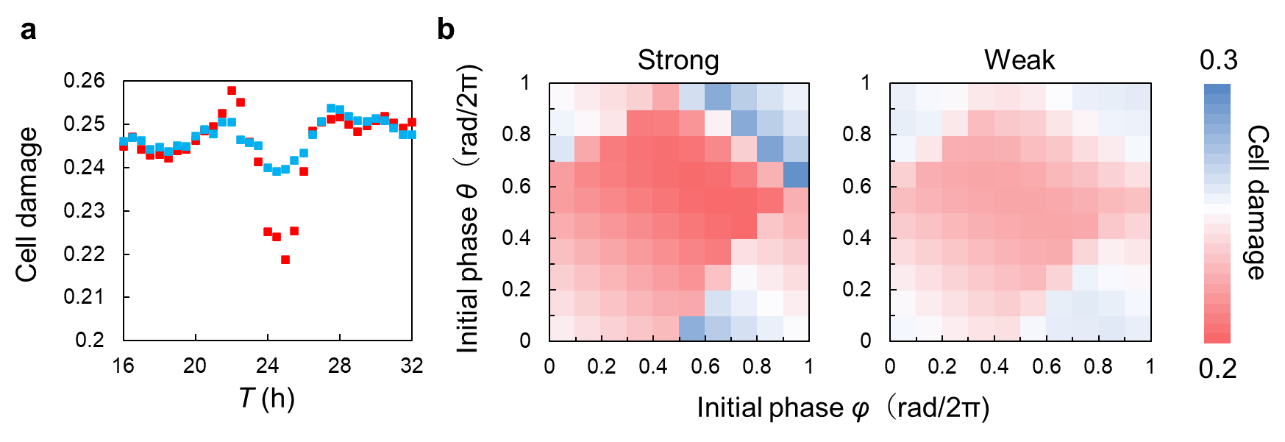


Figure S10. Effects of multicellularity on cell damage based on the phase of circadian clock with coupling (II). **a** Cell damage against the period of environmental cycles. **b** Cell damage for each initial phase with strong or weak intercellular couplings. The condition of couplings is the same as in Figure 7.


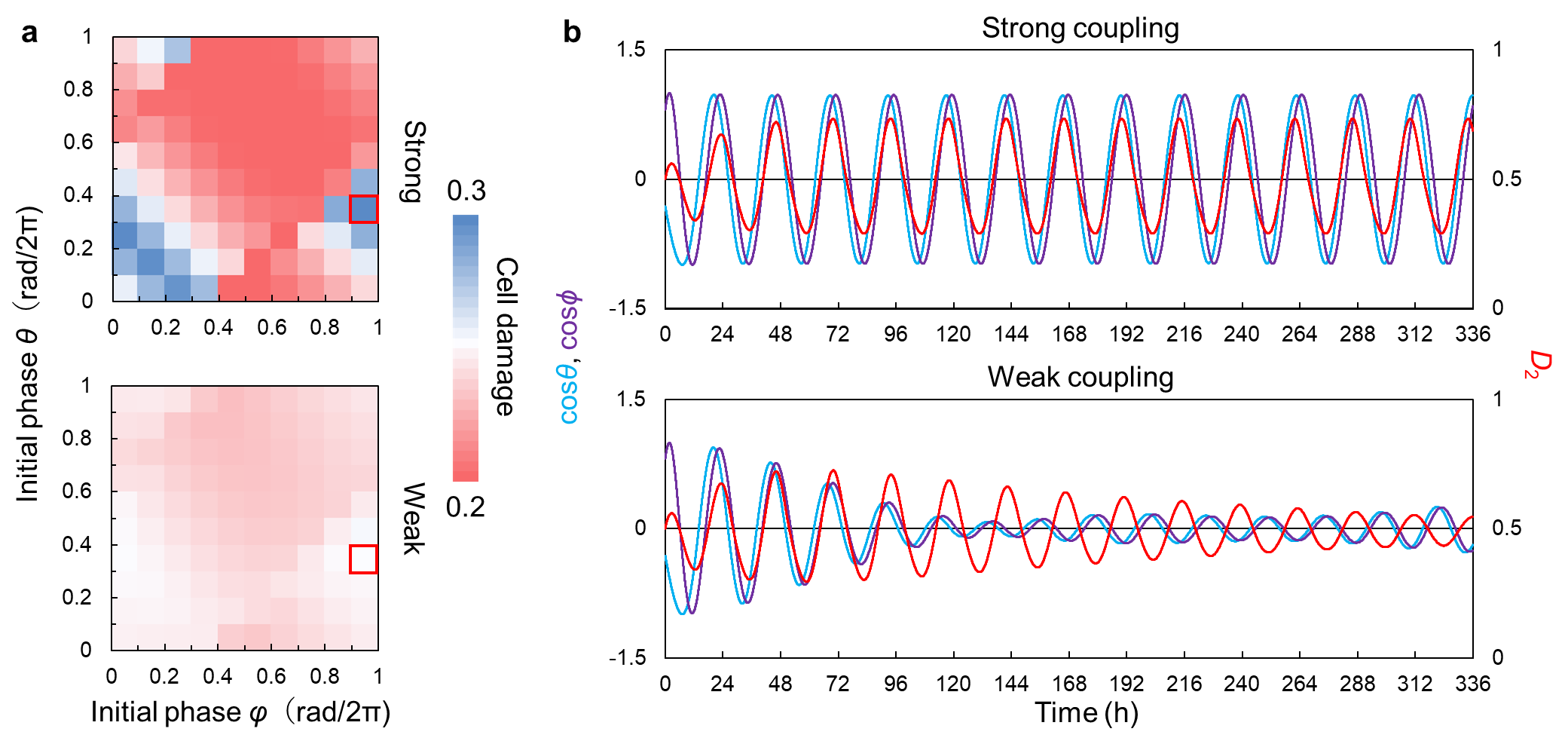


Figure S11. Effects of multicellularity on cell damage with coupling (III). **a** Cell damage for each initial phase with strong or weak intercellular couplings. **b** Circadian rhythms in strongly and weakly coupled population under the unsuitable initial phases (surrounded by red box in **a**). cos*θ* and cos*ϕ* are shown on the left axis, and *D*_2_ on the right axis.


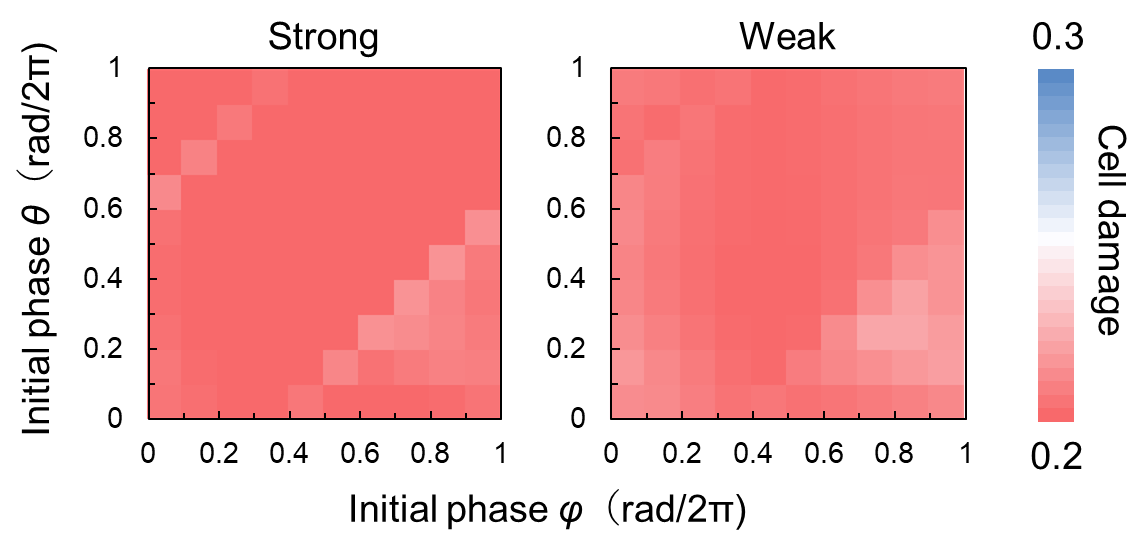


Figure S12. Effects of multicellularity on cell damage with coupling (IV) and strong or weak intercellular couplings for each initial phase.


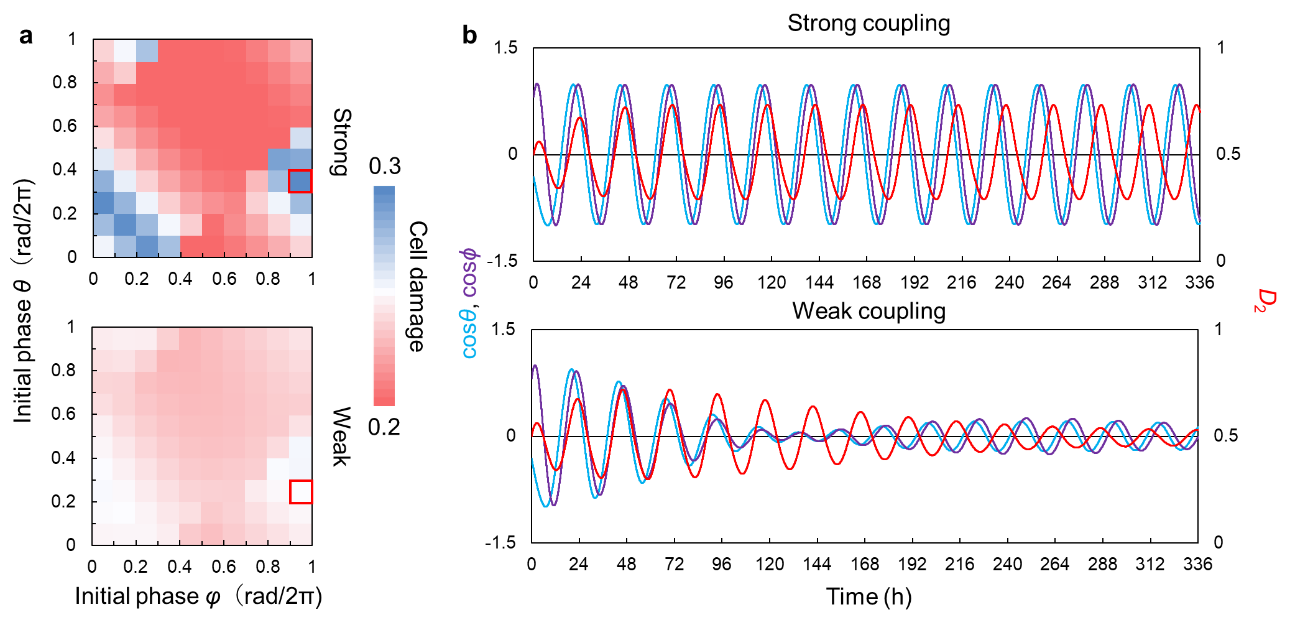


Figure S13. Effects of multicellularity on cell damage with coupling (V). **a** Cell damage for each initial phase with strong or weak intercellular couplings. **b** Circadian rhythms in strongly and weakly coupled population under the unsuitable initial phases (surrounded by red box in **a**). cos*θ* and cos*ϕ* are shown on the left axis, and *D*_2_ on the right axis.


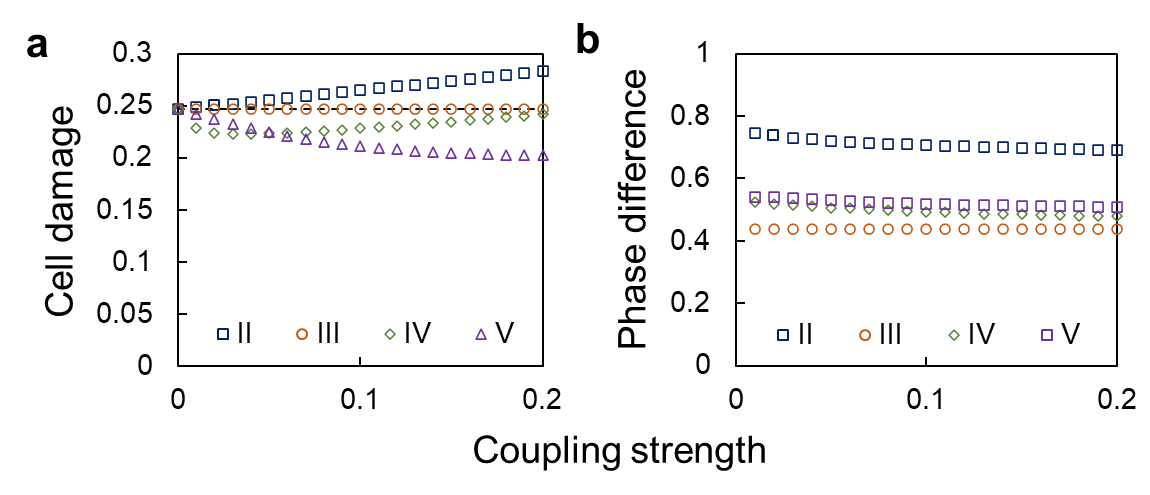


Figure S14. Unilateral coupling from the cell cycle to the circadian rhythm. **a** Average cell damages with the unilateral coupling (I) and other couplings. **b** Phase difference between cell cycle and redox rhythm with the unilateral coupling (I) and other couplings.


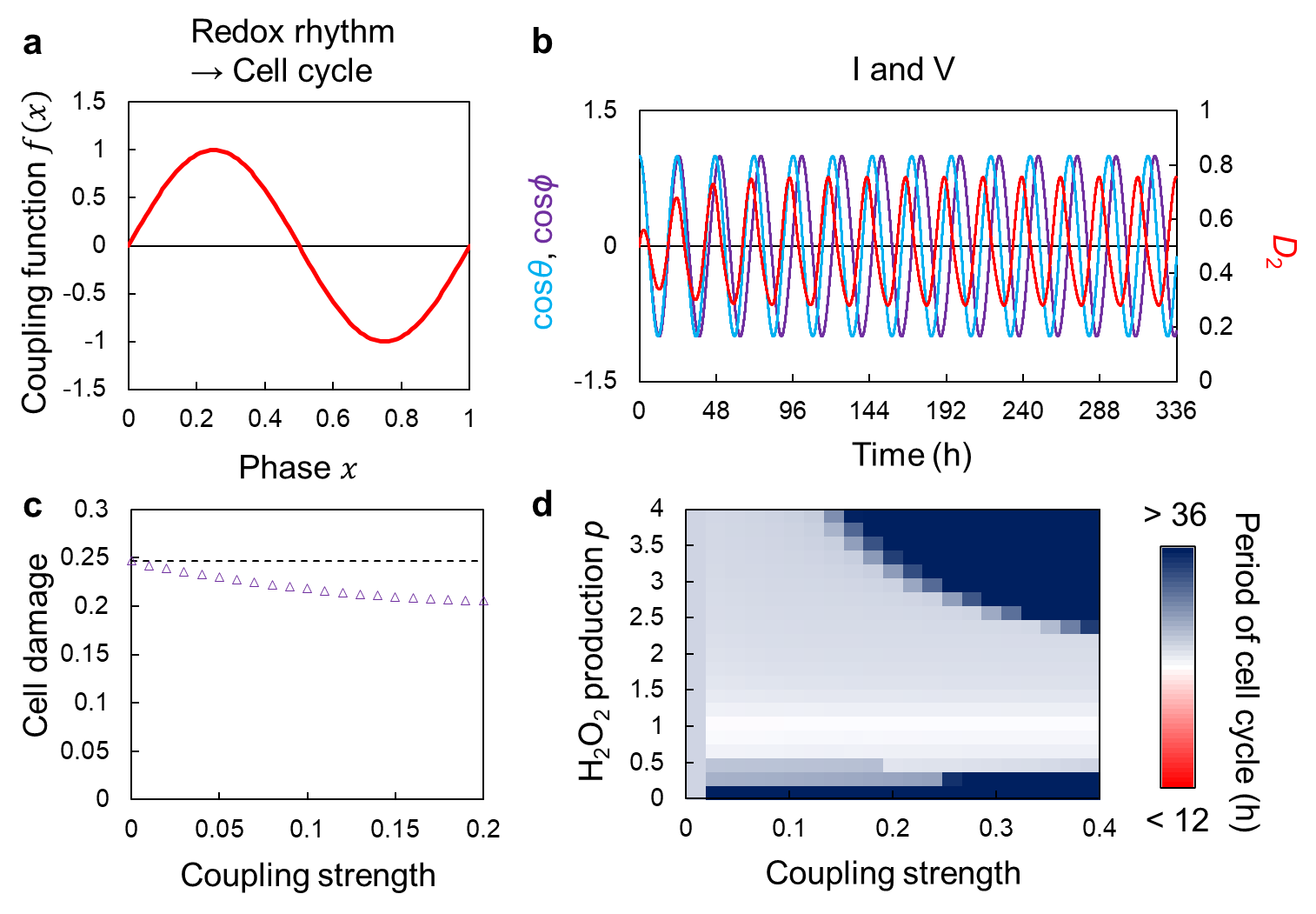


Figure S15. Sinusoidal coupling from redox rhythm to cell cycle. **a** Coupling function with a sinusoidal waveform. **b** Synchronization among three biological oscillators with sinusoidal coupling. cos*θ* and cos*ϕ* are shown on the left axis, and *D*_2_ on the right axis. **c** Cell damage against the coupling strength of coupling (V) with sinusoidal waveform. **d** Cell cycle arrest under high H_2_O_2_ concentration with coupling (V) with sinusoidal waveform.


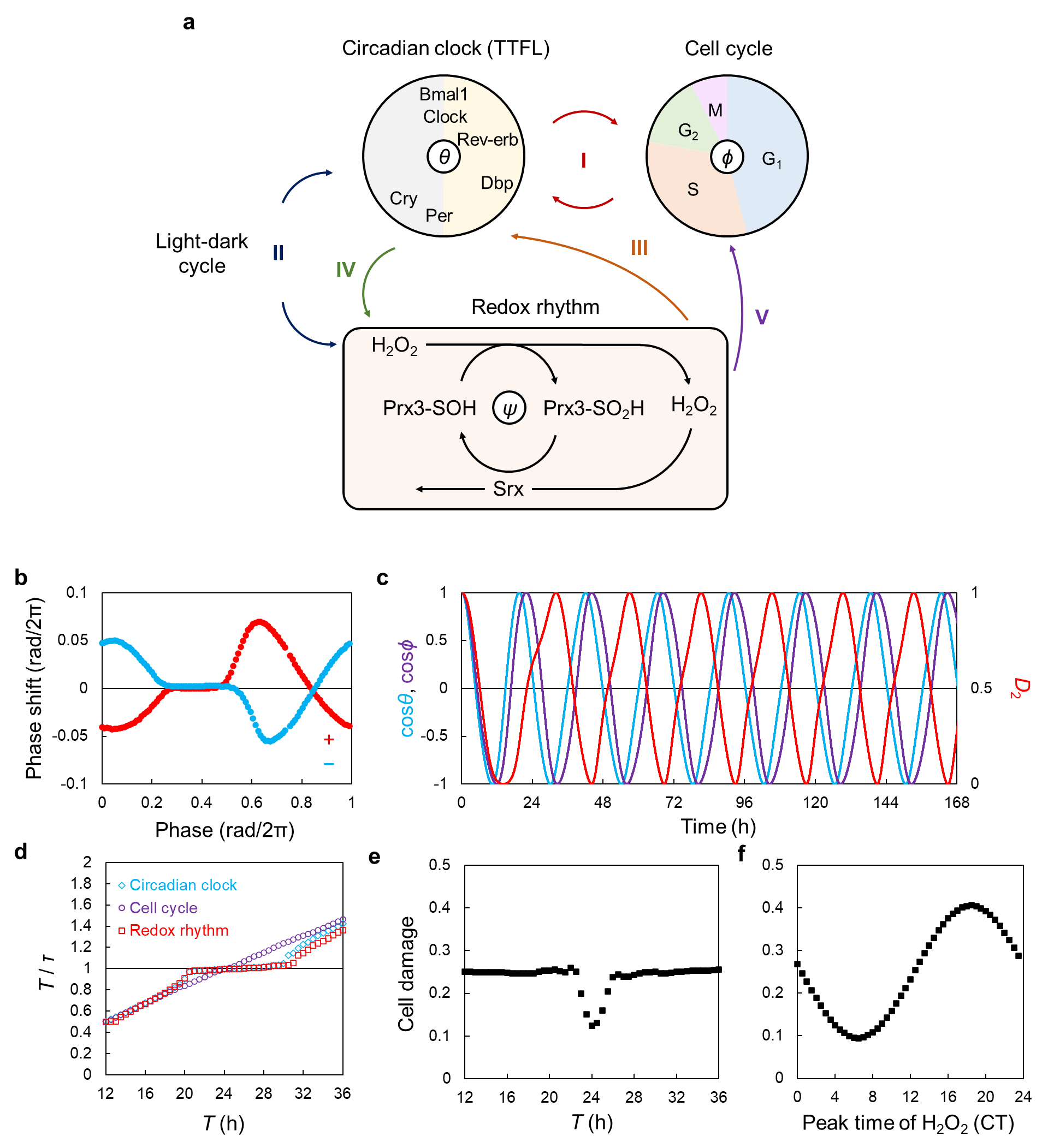


Figure S16. Phase reduction of redox rhythm. **a** Overview of the simplified model. Redox rhythm is represented by phase *ψ*. **b** PRC of redox rhythm to H_2_O_2_ stimuli (red for increase and blue for decrease of H_2_O_2_). **c** Three oscillator rhythms in the simplified model. cos*θ* and cos*ϕ* are shown on the left axis, and *D*_2_ on the right axis. **d** Synchronization of each rhythm with periodic stimuli. **e** Cell damages in different environmental periods. **f** Effect of the peak time of H_2_O_2_ production in the circadian time on cell damages.


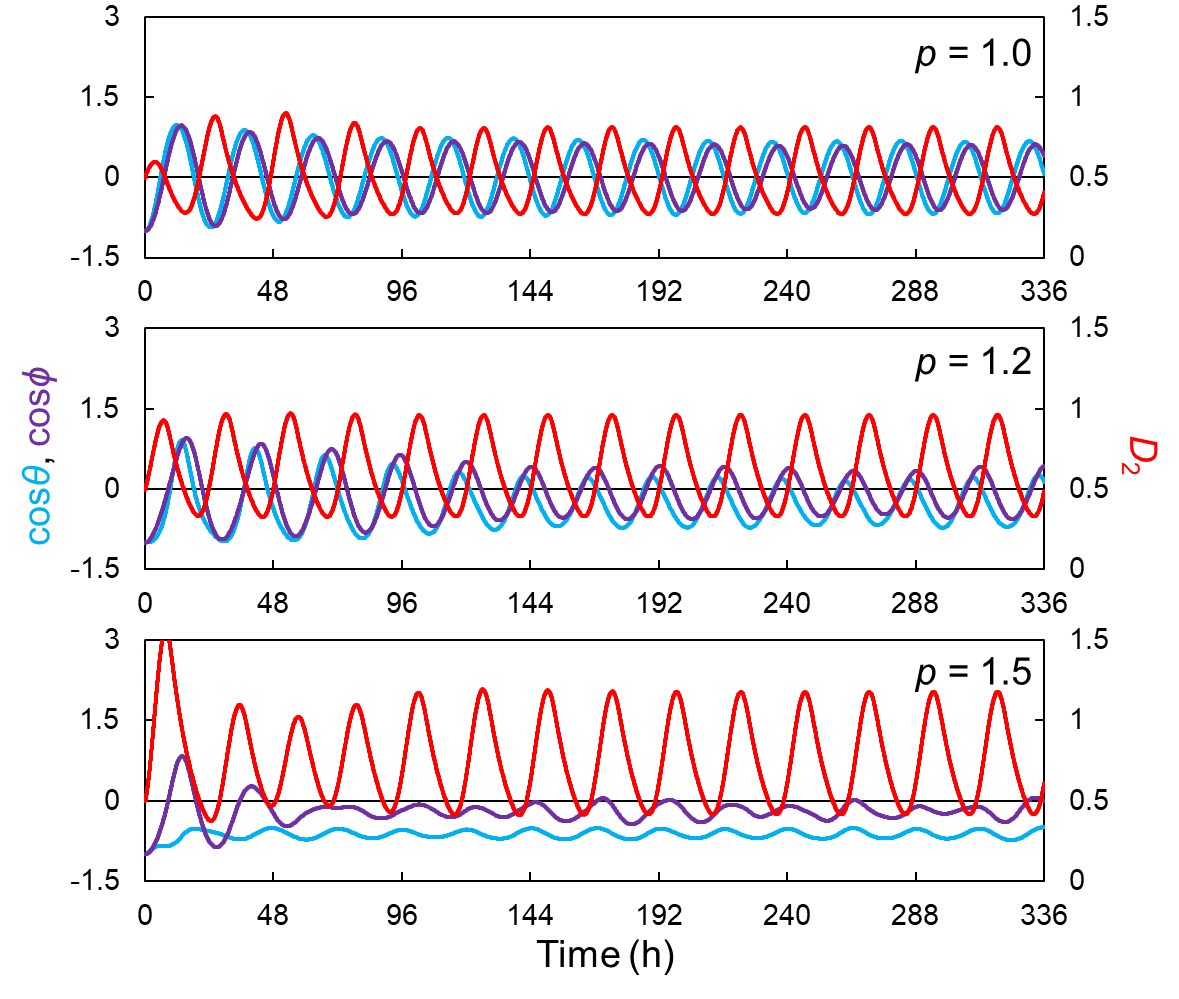


Figure S17. Population rhythms at high H_2_O_2_ concentration. *p* represents the average H_2_O_2_ production in mitochondria. *θ* = *ϕ* = π (rad) and *D*_1_ = *D*_2_ = *R* = 0.5 at *t* = 0 (h) in each condition. cos*θ* and cos*ϕ* are shown on the left axis, and *D*_2_ on the right axis.

Table S1. Coupling strengths in each condition.

|  | cir -> red | light -> cir | light -> red | red -> cir | cel -> cir | cir -> cel | red -> cel | cir -> cir | cel -> cel | red -> red |
| --- | --- | --- | --- | --- | --- | --- | --- | --- | --- | --- |
| Fig. 2 (Only I) | 0 | 0 | 0 | 0 | 0.01 | 0.01 | 0 |  |  |  |
| Fig. 2 (I and II) | 0 | 0.1 | 0.1 | 0 | 0.01 | 0.01 | 0 |  |  |  |
| Fig. 2 (I and III) | 0 | 0 | 0 | 0.2 | 0.01 | 0.01 | 0 |  |  |  |
| Fig. 2 (I and IV) | 0.1 | 0 | 0 | 0 | 0.01 | 0.01 | 0 |  |  |  |
| Fig. 2 (I and V) | 0 | 0 | 0 | 0 | 0.01 | 0.01 | 0.2 |  |  |  |
| Fig. 2 (All) | 0.1 | 0.1 | 0.1 | 0.2 | 0.01 | 0.01 | 0.2 |  |  |  |
| Fig. 3a, b | 0 | 0.1 | 0.1 | 0 | 0.01 | 0.01 | 0 |  |  |  |
| Fig. 3d | 0.1 | 0 | 0 | 0 | 0.01 | 0.01 | 0 |  |  |  |
| Fig. 3d | 0.1 | 0.1 | 0.1 | 0 | 0.01 | 0.01 | 0 |  |  |  |
| Fig. 4 | 0-0.2 | 0-0.2 | 0-0.2 | 0-0.2 | 0.01 | 0.01 | 0-0.2 |  |  |  |
| Fig. 5b, c (weak) | 0 | 0.3 | 0 | 0 | 0.01 | 0.01 | 0.1 |  |  |  |
| Fig. 5b, c (strong) | 0 | 0.3 | 0 | 0 | 0.1 | 0.1 | 0.1 |  |  |  |
| Fig. 6a | 0 | 0 | 0 | 0 | 0 | 0 | 0.3 |  |  |  |
| Fig. 6b | 0 | 0 | 0 | 0 | 0 | 0 | 0-0.4 |  |  |  |
| Fig. 7 (strong) | 0 | 0.05 | 0.05 | 0 | 0.1 | 0.1 | 0 | 0.1 | 0.1 | 0.1 |
| Fig. 7 (weak) | 0 | 0.05 | 0.05 | 0 | 0.1 | 0.1 | 0 | 0.01 | 0.01 | 0.01 |
| Fig. S2 | 0 | 0 |  | 0 | 0 | 0 | 0 |  |  |  |
| Fig. S3 | 0-0.2 | 0-0.2 | 0-0.2 | 0-0.2 | 0.01 | 0.01 | 0-0.2 |  |  |  |
| Fig. S4 (Only I) | 0 | 0 | 0 | 0 | 0.01 | 0.01 | 0 |  |  |  |
| Fig. S4 (I and II) | 0 | 0.1 | 0.1 | 0 | 0.01 | 0.01 | 0 |  |  |  |
| Fig. S4 (I and III) | 0 | 0 | 0 | 0.2 | 0.01 | 0.01 | 0 |  |  |  |
| Fig. S4 (I and IV) | 0.1 | 0 | 0 | 0 | 0.01 | 0.01 | 0 |  |  |  |
| Fig. S4 (I and V) | 0 | 0 | 0 | 0 | 0.01 | 0.01 | 0.2 |  |  |  |
| Fig. S4 (All) | 0.1 | 0.1 | 0.1 | 0.2 | 0.01 | 0.01 | 0.2 |  |  |  |
| Fig. S5 (I and II) | 0 | 0.1 | 0.1 | 0 | 0.01 | 0.01 | 0 |  |  |  |
| Fig. S5 (I and III) | 0 | 0 | 0 | 0.2 | 0.01 | 0.01 | 0 |  |  |  |
| Fig. S5 (I and IV) | 0.1 | 0 | 0 | 0 | 0.01 | 0.01 | 0 |  |  |  |
| Fig. S5 (I and V) | 0 | 0 | 0 | 0 | 0.01 | 0.01 | 0.2 |  |  |  |
| Fig. S6 | 0 | 0.1 | 0.1 | 0 | 0.01 | 0.01 | 0 |  |  |  |
| Fig. S7 | 0 | 0.1 | 0.1 | 0 | 0.01 | 0.01 | 0 |  |  |  |
| Fig. S8 (WT, without V) | 0.1 | 0 | 0 | 0.1 | 0.01 | 0.01 | 0 |  |  |  |
| Fig. S8 (KO, without V) | 0 | 0 | 0 | 0 | 0 | 0 | 0 |  |  |  |
| Fig. S8 (WT, with V) | 0.1 | 0 | 0 | 0.1 | 0.01 | 0.01 | 0.1 |  |  |  |
| Fig. S8 (KO, with V) | 0 | 0 | 0 | 0 | 0 | 0 | 0.1 |  |  |  |
| Fig. S9a | 0 | 0.1 | 0.1 | 0 | 0.01 | 0.01 | 0 |  |  |  |
| Fig. S9b | 0.1 | 0 | 0 | 0 | 0.01 | 0.01 | 0 |  |  |  |
| Fig. S9c | 0.1 | 0.1 | 0.1 | 0 | 0.01 | 0.01 | 0 |  |  |  |
| Fig. S9d | 0-0.2 | 0-0.2 | 0-0.2 | 0-0.2 | 0.01 | 0.01 | 0-0.2 |  |  |  |
| Fig. S10 (weak) | 0 | 0.05 | 0.05 | 0 | 0.1 | 0.1 | 0 | 0.1 | 0.1 | 0.1 |
| Fig. S10 (strong) | 0 | 0.05 | 0.05 | 0 | 0.1 | 0.1 | 0 | 0.01 | 0.01 | 0.01 |
| Fig. S11 (weak) | 0 | 0 | 0 | 0.1 | 0.1 | 0.1 | 0 | 0.1 | 0.1 | 0.1 |
| Fig. S11 (strong) | 0 | 0 | 0 | 0.1 | 0.1 | 0.1 | 0 | 0.01 | 0.01 | 0.01 |
| Fig. S12 (weak) | 0.05 | 0 | 0 | 0 | 0.1 | 0.1 | 0 | 0.1 | 0.1 | 0.1 |
| Fig. S12 (strong) | 0.05 | 0 | 0 | 0 | 0.1 | 0.1 | 0 | 0.01 | 0.01 | 0.01 |
| Fig. S13 (weak) | 0 | 0 | 0 | 0 | 0.1 | 0.1 | 0.1 | 0.1 | 0.1 | 0.1 |
| Fig. S13 (strong) | 0 | 0 | 0 | 0 | 0.1 | 0.1 | 0.1 | 0.01 | 0.01 | 0.01 |
| Fig. S14 | 0-0.2 | 0-0.2 | 0-0.2 | 0-0.2 | 0.01 | 0 | 0-0.2 |  |  |  |
| Fig. S15 | 0 | 0 | 0 | 0 | 0 | 0 | 0.3 |  |  |  |
| Fig. S16c | 0.05 | 0.1 | 0.1 | 0.1 | 0.01 | 0.01 | 0.1 |  |  |  |
| Fig. S16d, e | 0 | 0.1 | 0.1 | 0 | 0.01 | 0.01 | 0 |  |  |  |
| Fig. S16f | 0.05 | 0 | 0 | 0 | 0.01 | 0.01 | 0 |  |  |  |
| Fig. S17 | 0 | 0.05 | 0.05 | 0.1 | 0.1 | 0.1 | 0 | 0.01 | 0.01 | 0.01 |
